# A novel lncRNA promotes myogenesis of bovine skeletal muscle satellite cells via PFN1‐RhoA/Rac1

**DOI:** 10.1111/jcmm.16427

**Published:** 2021-05-04

**Authors:** Mingming Chen, Linlin Zhang, Yiwen Guo, Xinfeng Liu, Yingshen Song, Xin Li, Xiangbin Ding, Hong Guo

**Affiliations:** ^1^ Tianjin Key Laboratory of Agricultural Animal Breeding and Healthy Husbandry College of Animal Science and Veterinary Medicine Tianjin Agricultural University Tianjin China

**Keywords:** bovine, lnc23, myogenesis, PFN1, skeletal muscle satellite cells

## Abstract

Myogenesis, the process of skeletal muscle formation, is a highly coordinated multistep biological process. Accumulating evidence suggests that long non‐coding RNAs (lncRNAs) are emerging as a gatekeeper in myogenesis. Up to now, most studies on muscle development‐related lncRNAs are mainly focussed on humans and mice. In this study, a novel muscle highly expressed lncRNA, named lnc23, localized in nucleus, was found differentially expressed in different stages of embryonic development and myogenic differentiation. The knockdown and over‐expression experiments showed that lnc23 positively regulated the myogenic differentiation of bovine skeletal muscle satellite cells. Then, TMT 10‐plex labelling quantitative proteomics was performed to screen the potentially regulatory proteins of lnc23. Results indicated that lnc23 was involved in the key processes of myogenic differentiation such as cell fusion, further demonstrated that down‐regulation of lnc23 may inhibit myogenic differentiation by reducing signal transduction and cell fusion among cells. Furthermore, RNA pulldown/LC‐MS and RIP experiment illustrated that PFN1 was a binding protein of lnc23. Further, we also found that lnc23 positively regulated the protein expression of RhoA and Rac1, and PFN1 may negatively regulate myogenic differentiation and the expression of its interacting proteins RhoA and Rac1. Hence, we support that lnc23 may reduce the inhibiting effect of PFN1 on RhoA and Rac1 by binding to PFN1, thereby promoting myogenic differentiation. In short, the novel identified lnc23 promotes myogenesis of bovine skeletal muscle satellite cells via PFN1‐RhoA/Rac1.

## 1 INTRODUCTION

Myogenesis is an extremely complex and delicate process driven by many regulatory factors such as paired box families (Pax3/7), myogenic regulatory factors (Myogenin, MyoD, Myf5 and MRF4/6) and myocyte enhancer factor 2 (MEF2) family proteins^1^, ^2^, ^3^; these factors collectively regulate the expression of muscle‐specific genes and to control skeletal muscle development. Skeletal muscle satellite cells, as the stem cells of skeletal muscle, are located between skeletal muscle myofibre membrane and basal lamina membrane.^4^, ^5^ The activation, proliferation, migration, alignment, fusion and differentiation of skeletal muscle satellite cells to form contractible and beating multinucleate myotubes is very crucial for myogenesis.^6^, ^7^, ^8^ Increasing evidence indicating that non‐coding RNAs (ncRNAs) are widely involved in the process of myogenesis.^9^, ^10^

Long non‐coding RNAs (lncRNAs) are a class of RNA transcripts (>200 nt) with weak or no protein coding potential. With the mysterious functions of lncRNAs gradually be unveiled, accumulating evidence shed light that various lncRNAs regulate skeletal muscle myogenesis in a direct or indirect manner. LncRNAs can act as a molecular sponge to competitively bind microRNAs (miRNAs) or proteins to regulate skeletal muscle development. LncMD can act as competing endogenous RNA (ceRNA), competing with IGF2 and binding miR‐125b to weak its inhibitory effect on IGF2, promoting the differentiation of bovine skeletal muscle satellite cells.^11^ LncRNAs regulate skeletal muscle development by mediating epigenetic regulation. For example, MyoD induces lncRNA Dum, the upstream transcriptional product of Dppa2, to recruit a variety of DNA methyltransferase Dnmts to the CpG site of the Dppa2 promoter region, leading to the silencing of the neighbouring gene Dppa2 by cis action, and forming a MyoD‐Dum‐Dppa2 regulatory axis, thus positively regulating myoblast differentiation and muscle regeneration.^12^ LncRNAs also affect skeletal muscle development by regulating the splicing, stability and abundance of target mRNAs. For example, lncRNA Msx1 AS, transcribed from the anti‐sense chain of Msx1, interferes with the splicing process of pre‐mRNA and blocks the expression of Msx1 protein by binding to pre‐mRNA transcribed by Msx1, thus promoting myoblast differentiation.^13^ In addition, lncRNAs encode micropeptides to regulate skeletal muscle development. A research evinced that lncRNA‐Six1 can encode a 7.26 kD micropeptide, which is enriched in the promoter region of Six1 gene as a key regulatory factor, while lncRNA‐Six1 targets and cis‐regulates the expression of Six1 gene through the micropeptides and then positively regulates the proliferation and development of chicken muscle cells.^14^ It seems that lncRNAs are emerging as a gatekeeper of skeletal muscle development. However, most studies of lncRNAs focussed on humans and mice.^15^ Actually, although numerous lncRNAs have been detected in bovine skeletal muscle,^16^, ^17^, ^18^, ^19^ only a rare lncRNAs was identified as a molecular switch in bovine muscle development. Thus, to accelerate elucidating the mechanism in regulating bovine myogenic differentiation and provide a new target reference for cattle molecular breeding, we concentrated on the identification of bovine skeletal muscle‐related lncRNAs.

Profilin (PFN) is a highly conserved actin‐binding protein, which was widely found in eukaryotes and four subtypes have been found in mammals, including PFN1, PFN2, PFN3 and PFN4.^20^, ^21^, ^22^ PFN1 contains three functional domains, including actin‐binding domain, phosphatidylinositol‐4,5‐bisphosphate (PIP2) binding domain and poly‐l‐proline (PLP) binding domain.^23^, ^24^ Actin‐related protein‐2/3 complex (Arp2/3 complex) is a core participant in actin polymerization, and Arp2/3‐mediated actin polymerization plays an important role in myoblasts fusion.^25^ PFN1 competitively binds to filament barbed ends with capping protein, and PFN1 inhibits actin‐based cell movement mediated by Arp2/3 complex in a concentration‐dependent manner.^25^ By binding to its ligand PIP2, PFN1 inhibits the formation of inositol triphosphate (IP3) and diacylglycerol (DG) and prevents the release of Ca^2+^.^26^ At the same time, PIP2 and actin have the same binding site on PFN1, which results in PFN1 unable to form a complex with actin after binding to PIP2, further regulating actin polymerization/depolymerization and mediating cytoskeleton remodelling.^20^, ^27^ During myogenesis, a series of nuclear movement events occur in the nucleus of myoblasts, including centration, alignment, spreading, peripheral migration and anchoring, which mostly depend on microtubule (MT), kinesin and dynein motors.^28^ It reported that MTs, actin, and actin nucleators (formins and the Arp2/3 complex) compete for PFN1,^29^ suggesting that PFN1 is involved in myogenesis. Besides, actin filaments (F‐actin) formed by G‐actin polymerization is essential for myoblasts fusion to form myotubes.^30^ A wide range of cytoskeleton remodelling occurs before and after the fusion of mouse myoblasts.^31^ Without proper actin cytoskeleton remodelling, F‐actin accumulates between cell‐cell contact areas, resulting in a decrease in fused myoblasts.^30^ Therefore, PFN1 plays a critical role in skeletal muscle development. Specifically, PFN1 affects the remodelling of cytoskeleton to regulate myogenic differentiation by Arp2/3 mediated actin kinetics. And, PFN1 also functions in the transmission of intracellular signals to regulate myogenic differentiation via PIP2.

The small GTPase Rho family is a member of Ras superfamily. Human Rho subfamily includes more than 20 members such as RhoA, Rac1 and Cdc42, which are widely found in eukaryotic cells.^32^ As a molecular switch, small GTPase family members are widely involved in actin cytoskeleton assembly, cell adhesion, migration, proliferation and cell cycle, transcription factor activity and other biological processes.^32^ It has been confirmed that small GTPase Rho family is a key regulator of actin dynamics and plays a crucial role in regulating the dynamic changes of cytoskeleton.^33^, ^34^ For example, the activation of Rho, Rac and Cdc42 can induce cytoskeleton remodelling by regulating actin kinetics.^35^ RhoA, RhoB and RhoC can activate downstream ROCK to phosphorylate a series of actin cytoskeleton regulatory factors, and then regulate cytoskeleton remodelling and cell contraction.^36^ The downstream effect protein ROCK of RhoA can phosphorylate myosin and promote cell contraction, which is essential for cell proliferation.^37^ In addition, mDia, another RhoA downstream effect protein, can activate SKP2 and inhibit p27kip, resulting in a transition of G1/S phase.^38^ Meanwhile, RhoA and its downstream serum response factor (SRF) signalling pathway are also essential for skeletal muscle myogenesis. As an indispensable regulatory of MyoD expression, SRF is activated by RhoA and strongly promotes myogenic differentiation.^39^, ^40^ It is reported that Rac1 can promote cell fusion, while the absence of Rac1 strongly suppresses myoblasts fusion.^41^ In summary, small GTPase Rho family plays a key role in regulating the development of skeletal muscle.

In this study, we discovered and identified a novel highly expressed bovine muscle lncRNA–lnc23, which could promote the myogenic differentiation of bovine skeletal muscle satellite cells. Further studies demonstrated that PFN1 was a binding protein of lnc23, and we initially elucidated that lnc23 might regulate myogenic differentiation through affecting the role of PFN1 on RhoA/Rac1.

## 2 MATERIALS AND METHODS

### 2.1 Tissue sample collection and preparation

The tissue samples were collected from a cattle farm in Fengzhen, Inner Mongolia, and met the national feeding standard NT/T815‐2004. All procedures performed for this study were consistent with the National Research Council Guide for the Care and Use of Laboratory Animals. All animal experimental protocols were approved by the Institute of Animal Science of Tianjin Agricultural University and were carried out in strict ethical policy. All cattle had not experienced any diseases. Specifically, four types of skeletal muscle samples (scapular muscle, gluteus muscle, longissimus muscle and intercostal muscle) and other non‐skeletal muscle samples (heart, liver, spleen, lung, kidney, small intestine and stomach) of Inner Mongolian black cattle were collected from 3‐month‐old foetus, 6‐month‐old foetus and 9‐month‐old neonatal calf. All samples were immediately frozen in liquid nitrogen and then stored at −80°C until analysis. And the gluteus muscle, scapular muscle, intercostal muscle and longissimus muscle from 3‐month‐old foetus, 6‐month‐old foetus and 9‐month‐old neonatal calf were selected for high‐throughput sequencing approaches.

### 2.2 Cell isolation and culture

Bovine skeletal muscle satellite cells were isolated and cultured as previously described.^42^ In brief, the muscle tissues of the hind limbs from 5‐ to 6‐month‐old bovine foetuses were cut into small pieces, digested with 0.2% collagenase type II (Gibco, Grand Island, NY) at 37°C for 1 hour and then centrifuged at 2000 × *g* for 10 minutes. The precipitates were continue digested with 0.25% trypsin (Gibco, Grand Island, NY) at 37°C for 30 minutes, and digestion was terminated with serum containing medium. Cell suspension was successively filtered through 100, 200 and 400 mesh cell sieves. After this, the cells were centrifuged at 2000 × *g* for 10 minutes, resuspended in growth medium (GM) containing Dulbecco's modified Eagle's medium (DMEM/High Glucose, Hyclone, Logan, Utah) with 20% foetal bovine serum (FBS, Gibco) and 1% penicillin‐streptomycin liquid (Solarbio, Beijing, China) and cultured for 2‐3 times with differential adhesion. To induce differentiation, the cells were cultured to 70% confluence in GM and followed by an exchange to differentiation medium (DM) containing DMEM with 2% horse serum (HS, Gibco) and 1% penicillin‐streptomycin liquid to culture 24 hours, 48 hours and 72 hours.

### 2.3 Total RNA isolation and quantitative real‐time PCR (qRT‐PCR)

Total RNA of tissues and cells was isolated using TRIzol reagent (Invitrogen, USA) following the manufacturer's protocol. In short, after tissues or cells were lysed, chloroform was added to separate the organic and inorganic phases, followed by precipitation with isopropanol and ethanol in turn, and finally the RNA was dissolved in DEPC water. Then, the first strand cDNA was prepared using PrimeScript II 1st Strand cDNA Synthesis Kit (Takara, Dalian, China). qRT‐PCR was performed using All‐in‐One™ qRT‐PCR Mix (Genecopoeia, Guangzhou, China) in a LightCycler^®^ 96 Instrument (Roche, Germany). With GAPDH mRNA as an endogenous control, the relative expression level of genes was calculated by the 2^−ΔΔCt^ method. All primers used were listed in Table S1.

### 2.4 Rapid amplification of cDNA ends (RACE)

5′ and 3′ RACE experiments were performed to obtain the full length of lnc23 using the SMARTer RACE cDNA Amplification Kit (Clontech, USA) according to the manufacturer's protocol. Total RNA was isolated from bovine skeletal muscle myotubes that had been differentiated for 2 days (DM2). The gene‐specific primers used for RACE were listed in Tables S2 and S3.

### 2.5 Generation of constructs and siRNA synthesis

The sense and anti‐sense chains of lnc23 were, respectively, subcloned into the EcoRV and XhoI sites of pcDNA3.1(+) vector (Miaoling Bio, Wuhan, China) to obtain the recombinant expression plasmids pcDNA‐lnc23(+) and pcDNA‐lnc23(−). To construct plasmids for in vitro translation, the sense strand of lnc23 was subcloned into the NheI site of pET‐28a vector (Miaoling Bio, Wuhan, China) by seamless cloning; and the bovine *FSH‐β*, as a positive control, was subcloned into pET‐28a vector by EcoRI and HindIII restriction enzyme. All the primers sequences of gene cloning were listed in Table S4. All the siRNAs for gene knockdown and negative control (si‐NC) were synthesized by RiboBio (Guangzhou, China), and the sequences were listed in Table S5.

### 2.6 In vitro translation

Constructed recombinant plasmids pET‐lnc23 and pET‐FSH‐β were, respectively, transformed into BL21 competent cells, then single clones were selected to 1.5 mL liquid medium and incubated at 37°C 200 rpm until the optical density value (600 nm) reached 0.6‐0.8. Next, isopropyl β‐d‐thiogalactoside (IPTG) was added to a final concentration of 0.5 mmol/L and induced to culture for 2‐4 hours at 37°C 200 rpm. The samples were centrifuged at 18 000 × *g* for 1 minutes, the sediments were suspended in 50‐100 μL 10 mmol/L Tris‐HCl (pH 8.0) and the supernatants were subjected to SDS‐PAGE.

### 2.7 Cell transfection

According to the manufacturer's protocol, the cells were transfected with siRNA or plasmid using Lipofectamine 3000 (Invitrogen, USA) when they were cultured to 60%‐70% confluence in 96‐well, 24‐well or 6‐well plates. In all cell culture plates, the final concentrations used for siRNA and plasmid, respectively, were 100 nmol/L and 2.0 µg/mL. Finally, cells were collected for detection after transfection 24 hours and 72 hours.

### 2.8 Western blot analysis

Bovine skeletal muscle cells were lysed in RIPA buffer (Solarbio, Beijing, China) supplemented with PMSF for total protein characterization. Then, equal amounts of cells lysate were resolved by 10% or 12.5% SDS‐PAGE and transferred onto PVDF membranes (Millipore, USA). The membranes were blocked with 5% BSA for 1 hour, incubated with primary antibody at 4°C overnight and then incubated with secondary antibody for 1 hour before detection. The fold change of protein was normalized to GAPDH for quantitative analysis by ImageJ software. The antibodies information was listed in Table S6.

### 2.9 Nuclear and cytoplasmic fractionation

According to the previously described,^43^ the cytoplasmic and nuclear extracts from myoblasts and myotubes were stepwise separated using NE‐PER Nuclear and Cytoplasmic Extraction Reagents (Thermo Scientific, USA). In brief, the ice‐cold CER I with RNase inhibitor was used to break cell membranes and release cell contents. After centrifugation, the supernatant, which is the cytoplasmic extract, was transferred to a clean pre‐chilled tube to obtain cytoplasmic RNA. Then, the precipitate was washed 2‐3 times with PBS to obtain nuclear RNA.

### 2.10 Immunofluorescence staining

Bovine skeletal muscle cells were fixed in 4% paraformaldehyde for 30 minutes, permeabilized in 0.1% Triton X‐100 for 20 minutes and blocked with 5% normal goat serum for 30 minutes at room temperature, and incubated with primary antibody at 4°C overnight. Next, the FITC‐conjugated secondary antibody was added and incubated at 37°C for 1 hour in the dark, and the nuclear was then counterstained with DAPI. The immunofluorescence images from five random fields were visualized and captured with an inverted fluorescence microscope at a magnification of 200×.

### 2.11 TMT 10‐plex labelling quantitative proteomics

A total of 5 biological replicates were, respectively, set for two groups, group si‐ASO‐lnc23 and group si‐NC. The cells were induced differentiation for 2 days after transfection, then lysed in RIPA buffer (1× PBS (pH 7.4), 0.5% Sodium deoxycholate, 1.0% NP‐40 and 1.0% SDS solution) supplemented with PMSF. Then, the TMT 10‐plex labelling quantitative proteomics was performed to identify the difference proteins between the group si‐ASO‐lnc23 and group si‐NC, and the potential regulatory protein of lnc23 was screened using Perseus 1.5.3.2 software. Group si (si‐1‐si‐5) was, respectively, labelled by tags 126, 127C, 127N, 128C and 128N, while Group NC (NC‐1‐NC‐5) was, respectively, labelled by tags 129C, 129N, 130C, 130N and 131.

### 2.12 In vitro transcription

The recombinant plasmid pcDNA‐lnc23(+) and pcDNA‐lnc23(−) were linearized by XhoI restriction enzyme to prepare the templates with T7 promoter for in vitro transcription. Biotin‐labelled sense and anti‐sense chains of lnc23 were transcribed in vitro using biotin‐16‐UTP (Epicentre, USA) by T7 RNA polymerase. Then, the DNA template in the system was eliminated with DNase I, followed by RNA purification and electrophoresis detection.

### 2.13 RNA pulldown/LC‐MS

The Pierce™ Magnetic RNA‐protein Pull‐Down Kit (Thermo Scientific, USA) was utilized to capture potential binding protein of lnc23. The biotin‐labelled sense and anti‐sense chains of lnc23 were pretreated with RNA structure buffer (10 mmol/L Tris‐HCl pH 7.0, 0.1 mol/L KCl, 10 mmol/L MgCl_2_), and then incubated with RNA capture buffer, magnetic beads and 1 U/mL RNase inhibitor for 2 hours at 4°C. Next, the bovine skeletal muscle cells from DM2 were lysed using IP lysis buffer supplemented with 1× protease inhibitor cocktail, incubated with RNA‐beads complex for 2 hours at 4°C. Subsequently, the RNA‐Protein‐beads complex was washed five times, mixed with protein loading buffer and incubated at 100°C for 10 minutes. Finally, the supernatants were subjected to SDS‐PAGE and LC‐MS identification. The identified proteins were screened by protein contents in the molar fraction percentage.

### 2.14 RNA immunoprecipitation (RIP)

According to the manufacturer's protocol, RIP was performed using Magna RIP™ RNA‐Binding Protein Immunoprecipitation Kit (Sigma‐Aldrich, USA). In brief, the bovine skeletal muscle cells from DM2 were lysed using complete RIP lysis buffer supplemented with 1× protease inhibitor cocktail and 1 U/mL RNase inhibitor. The pretreated magnetic beads protein A/G and the primary antibody or IgG were incubated for 30 minutes at room temperature. Then, the beads‐antibody complex and cell lysates were incubated with rotating at 4°C overnight. The co‐precipitated RNAs were extracted with TRIzol reagent.

### 2.15 Statistical analysis

All results are presented as the mean ± SEM. Statistical analyses of differences between groups were performed using two‐tailed Student's *t* test or chi‐square test and *P* < .05 was considered statistically significant. *P < .05 and **P < .01. Protein content in the molar fraction percentage^44^ was calculated by the following formula:

$$\text{Protein content}\;\left(\text{mol}\%\right)=\frac{\text{emPAI}}{\sum \left(\text{emPAI}\right)}\times 100$$

## 3 RESULTS

### 3.1 Identification, characterization and expression pattern of lnc23

To enrich and expand the understanding of lncRNAs in bovine skeletal muscle development, the gluteus muscle, scapular muscle, intercostal muscle and longissimus muscle from three representative embryonic development periods, 3‐month‐old foetus, 6‐month‐old foetus and 9‐month‐old neonatal calf were selected and performed high‐throughput sequencing (Data not published). A total of 63 509 lncRNAs were identified, of which 180 lncRNAs were specifically co‐expressed in muscle tissues of three representative periods under the condition of adjusted *P*‐value <.05 (Figure S1A). Then, the cluster analysis of 180 co‐expressed lncRNAs showed that the expression of lncRNAs was different in the three periods, indicating that these lncRNAs might play an important role in bovine muscle development (Figure S1B). Considering the low abundance characteristic of lncRNAs, the standard of RPKM > 5 in each period was used for filtering and 4 lncRNAs were screened (Figure S1A). Among them, there are three bases exactly overlapping between the 3′ end of TCONS_00137442 and the 5′ end of TCONS_00139622. After splicing them together, it was found that it can be completely aligned to chromosome 23 of the bovine genome. Therefore, TCONS_00137442 and TCONS_00139622 were considered to be the same lncRNA and named lnc23.

**FIGURE 1:**
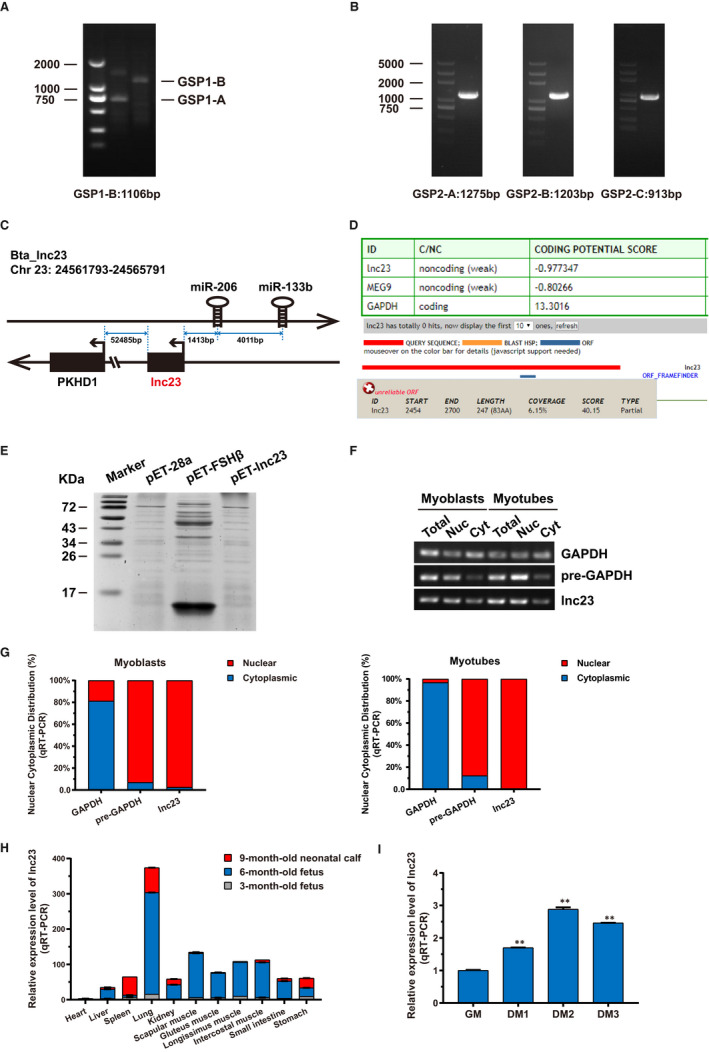
Characterization and expression pattern of lnc23. (A) 5′‐RACE of lnc23 in bovine skeletal muscle cells. GSP1‐A and GSP1‐B are the gene‐specific primers (GSPs) of 5′‐RACE. (B) 3′‐RACE of lnc23 in bovine skeletal muscle cells. GSP2‐A, GSP2‐B and GSP2‐C are the gene‐specific walking primers of 3′‐RACE. (C) Genome localization schematic of lnc23. Lnc23 is located on chromosome 23 of the bovine genome, the upstream genes are miR‐133b and miR‐206, and its downstream gene is PKHD1. (D) Prediction of coding potential using CPC Program. Both lnc23 and MEG9 were predicted to be non‐coding RNA and found that lnc23 had a small open reading frame (ORF), while GAPDH was identified to code for protein. (E) In vitro translation system showed no evidence of protein product from lnc23, while the positive control bovine FSH‐β could encode a ~14 kD protein. (F‐G) The subcellular location of lnc23. Total RNA, nuclear RNA and cytoplasmic RNA were extracted from proliferation cells (Myoblasts) and differentiated cells of 2 d (Myotubes). With the pre‐GAPDH and GAPDH, mRNAs were, respectively, used as nuclear and cytoplasmic iconic gene, the RT‐PCR (F) and qRT‐PCR (G) showed that lnc23 was predominantly located in nucleus. (H) Tissue expression profile of lnc23. The qRT‐PCR was performed to detect the expression of lnc23 in heart, liver, spleen, lung, kidney, scapular muscle, gluteus muscle, longissimus muscle, intercostal muscle, small intestine and stomach, of which were all from 3‐mo‐old foetus, 6‐mo‐old foetus and 9‐mo‐old neonatal calf. (I) Time series expression profile of lnc23. The bovine myoblasts and myotubes from GM, DM1, DM2 and DM3 were used for detecting lnc23 expression by qRT‐PCR

5′ and 3′ rapid amplification of cDNA ends (RACE) were performed to identify the full‐length sequence of lnc23. For 5′‐RACE, the extended 1106 bp by the primer GSP1‐A was determined to be the 5′ terminal sequence of lnc23 (Figure 1A). For 3′‐RACE, no fragments could be amplified by SMARTer method. And three pairs walking primers also failed to amplify the complete sequence at 3′ end of lnc23, although it has extended 3397 bp (Figure 1B). Then, the 5′ and 3′‐RACE results were spliced and obtained a 4001 nt fragment, which could be completely aligned to chromosome 23. Thus, we only carried out subsequent studies with the obtained lnc23 fragment of 4001 nt in length (GenBank Accession ID: BankIt2343098 bta_lnc23 MT721869). Genome localization analysis showed that lnc23 is located on chromosome 23 of the bovine genome, the upstream genes are miR‐133b and miR‐206, and its downstream gene is PKHD1 (Figure 1C). Some lncRNAs have the ability to encode micropetides. Therefore, the coding potential calculator (CPC) online tool (http://cpc.cbi.pku.edu.cn/) was used to predict the coding potential of lnc23. The result showed that lnc23 had low coding potential (−0.977347) and a small open reading frame (ORF) (Figure 1D). Then, an in vitro translation experiment was performed to ascertain this prediction. Results showed no evidence of protein product from lnc23, while the positive control bovine FSH‐β was proved as a protein coding gene with encoding a ~14 kD protein (Figure 1E), which further supported its non‐coding property. To determine the subcellular location of lnc23, total RNA, nuclear RNA and cytoplasmic RNA were extracted from bovine satellite cell‐derived myoblasts and myotubes. With the GAPDH and pre‐GAPDH mRNA were, respectively, as cytoplasmic and nuclear iconic gene, the RT‐PCR and qRT‐PCR suggested that lnc23 was all predominantly located in nucleus of myoblasts and myotubes (Figure 1F,G).

From 11‐12 weeks of the embryo (~3 months old) to birth, the tissues and organs of vertebrates are formed and further differentiated, and the foetal growth reaches its peak during this stage. Therefore, the tissue expression profiles at different developmental stages were performed to reveal whether lnc23 was implicated in myogenesis. Specifically, the expression of lnc23 was detected by qRT‐PCR in various tissues from 3‐month‐old foetus, 6‐month‐old foetus and 9‐month‐old neonatal calf. Results showed that the expression of lnc23 was prominently high in the muscle tissues of 6‐month‐old foetus, while lowly expressed in 3‐month‐old foetus and 9‐month‐old neonatal calf, that is lnc23 was highly expressed at the peak of cattle foetal growth and development, suggesting that lnc23 may participate in the regulation of skeletal muscle development and muscle formation in cattle (Figure 1H). To gain more insights, the expression level of lnc23 during myogenesis was assessed by qRT‐PCR. Results demonstrated that the expression of lnc23 is time‐dependent and reached the peak on the 2 days of induced differentiation (DM2) (Figure 1I), suggesting that lnc23 may have a role in the regulation of myogenesis.

### 3.2 Lnc23 promotes the differentiation of bovine myoblasts

**FIGURE 2:**
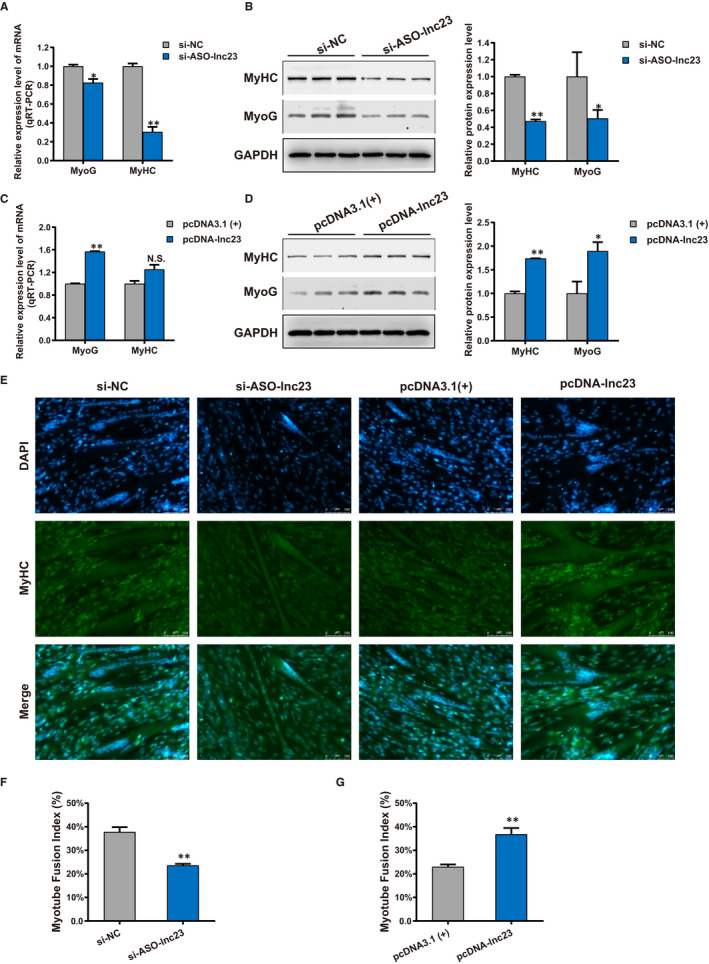
Lnc23 promotes the differentiation of bovine myoblasts. Knockdown experiment of lnc23 resulted in a decrease in mRNA (A) and protein (B) expression level of cell differentiation‐related markers MyoG and MyHC. (C‐D) While, the over‐expression of lnc23 increased the mRNA and protein expression level of MyoG and MyHC. (E) The MyHC immunofluorescence staining of myotubes in DM2 showed that the number of myotubes was reduced and increased when lnc23 was knocked down and over‐expressed (scale bars 100 μm), (F‐G) and the quantitative myotube fusion index has the same results

To unveil the role of lnc23 in myogenesis of bovine skeletal muscle satellite cells, nuclear specific interference RNA si‐ASO‐lnc23 was designed and synthesized for lnc23 knockdown, and the recombinant plasmid pcDNA‐lnc23 was constructed for lnc23 over‐expression, which successfully constructed the lnc23 loss‐of‐function and gain‐of‐function model with 83% decrease and more than 17 367‐fold increase of lnc23 expression level in GM, and produced 35% reduction and more than 1144‐fold increase of lnc23 expression level in DM2, respectively (Figure S2A,B). The results showed that knockdown of lnc23 inhibited the differentiation process of bovine skeletal muscle satellite cells, while over‐expression of lnc23 promoted differentiation process of bovine skeletal muscle satellite cells (Figure S2C). Hence, we speculated that lnc23 plays an important role in the differentiation of bovine myoblasts. To ascertain ours posit, the qRT‐PCR and Western blot were employed and exhibited that down‐regulated lnc23 resulted in a drastic decrease in mRNA (Figure 2A) and protein (Figure 2B) expression level of cell differentiation‐related markers MyoG and MyHC in DM2. On the contrary, over‐expression of lnc23 brought about a significant elevation of mRNA (Figure 2C) and protein (Figure 2D) expression level of MyoG and MyHC in DM2. This suggests that lnc23 may function in the cell differentiation of bovine myoblasts. To further verify the effect of lnc23 on cell differentiation, the myotubes MyHC immunofluorescence staining was performed in DM2. Results demonstrated that the number of myotubes (Figure 2E) and quantitative myotube fusion index (Figure 2F) were reduced when lnc23 was knocked down, while the reciprocal experiment gave rise to the opposite results (Figure 2E,G). As anticipated, lnc23 has been confirmed to play a key role in myogenic differentiation. Taken together, our experiments suggested that lnc23 positively regulates the differentiation of bovine myoblasts to involve in skeletal muscle myogenesis.

### 3.3 Quantitative proteomics analysis of lnc23 potentially regulated proteins

**FIGURE 3:**
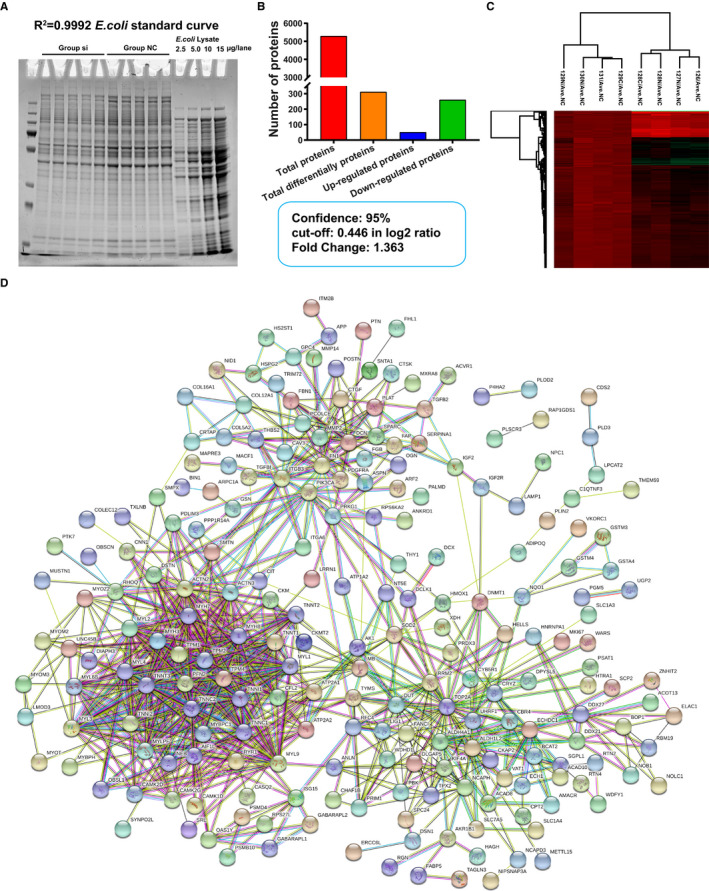
Analysis of TMT 10‐plex labelling quantitative proteomics. (A) Colloidal coomassie stain of samples. (B) The statistics of total proteins and differential expressed proteins (DEPs) were screened by Student's *t* test (*P* < .05). (C) Cluster analysis of 312 DEPs by Perseus 1.5.3.2 software. All data were normalized using the average of the group NC (Ave.NC). The group si and group NC were clustered into one group, respectively. (D) Protein‐protein interaction (PPI) analysis of DEPs by STRING

As aforementioned, lnc23 plays an essential role in regulating the differentiation of bovine skeletal muscle satellite cells. To further elucidate its regulation mechanism, obtain the information of potential regulatory proteins of lnc23 and shed light lnc23‐related regulatory pathway, a TMT 10‐plex labelling quantitative proteomics analysis was carried out on the basis of knocking down the expression of lnc23. Total proteins of group si‐ASO‐lnc23 and group si‐NC in DM2 were qualitatively and quantitatively analysed by colloidal Coomassie staining. Results showed that the total protein bands of all samples were clear and evenly distributed, and the mass was more than 100 μg, which met the requirements of proteomics identification (Figure 3A).

A total of 5295 proteins were identified by quantitative proteomics (Figure 3B), and each protein containing at least one unique peptide. Quantitative proteomics data were analysed by Perseus 1.5.3.2 software. In order to make the samples obey normal distribution, 127C tagged si‐2 and 130C tagged NC‐3 were excluded, respectively. Firstly, all the data were converted by log2, and two‐sample *t* test was performed after filtering out the invalid values. Among them, 1134 proteins were detected by *t* test, that is, 1134 proteins were differently expressed (*P* < .05) between group si‐ASO‐lnc23 and group si‐NC. With the ratio of si‐ASO‐lnc23/si‐NC as the Fold Change, and the cut‐off value of 0.446 as a screening criterion for further differentially expressed proteins (DEPs) at 95% confidence, thus all proteins with Fold Change > 1.363 were significantly up‐regulated while all proteins with Fold Change < 0.734 were significantly down‐regulated. Finally, a total of 312 DEPs were screened, including 51 significantly up‐regulated proteins and 261 significantly down‐regulated proteins (Figure 3B), which were regarded as potential regulatory proteins of lnc23. Then, the cluster analysis of 312 DEPs revealed that all samples were clustered into two groups, si‐ASO‐lnc23 and si‐NC, indicating that the samples were homogeneous and the data were neat (Figure 3C). In addition, 18 proteins with a large difference and two down‐regulated proteins, KBTBD10 and TPM1, were selected from 312 DEPs to verify the reliability of quantitative proteomics at mRNA and protein level, respectively. The results were consistent with quantitative proteomics (Figure S3), suggesting that proteomics results were accurate and reliable, and can be used as an important reference for future research.

**FIGURE 4:**
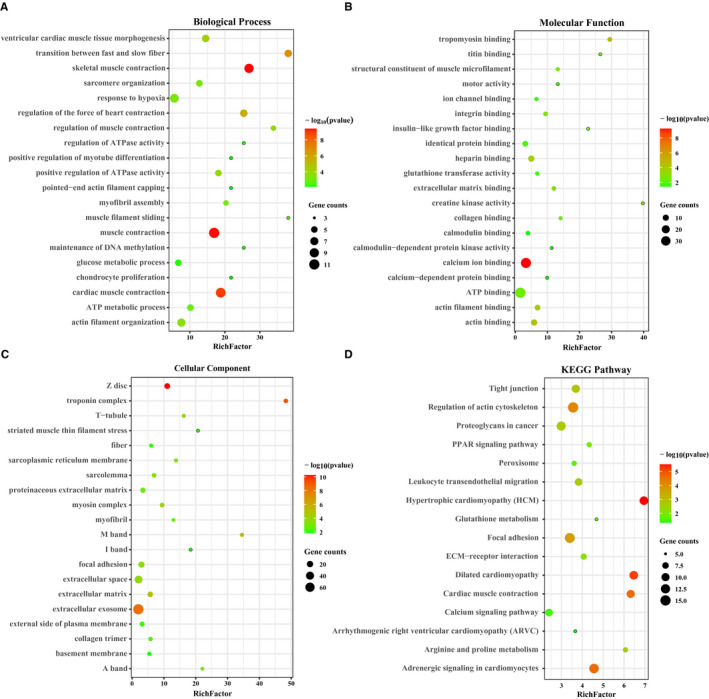
GO and KEGG analysis of differential expressed proteins. GO and KEGG analysis of differential expressed proteins were performed using DAVID. The enrichment items or pathways were filtered with *P* < .05. Only the top 20 items or pathways with the most significant enrichment were displayed using R 3.63 software

Protein‐protein interaction (PPI) analysis of 312 DEPs by STRING database revealed four major protein‐protein interaction networks (Figure 3D), including the muscle contraction‐related proteins; cell adhesion, cell motility, fibre formation, extracellular matrix and other cell fusion‐related proteins; ubiquitination, methylation and other protein modification‐related proteins; and RNA binding‐related proteins (Figure 3D). Among them, most of muscle contraction and cell fusion‐related proteins were down‐regulated, while most of protein modification and RNA binding‐related proteins were up‐regulated. PPI analysis suggesting that down‐regulated lnc23 might inhibit myogenic differentiation by attenuating signal transmission and cell fusion between cells and cells or extracellular matrix. This further demonstrated the key regulatory role of lnc23 in myogenic differentiation. To further explore the function of 312 DEPs and their regulation pathways, GO and KEGG analysis were performed by DAVID. GO enrichment analysis showed that 312 DEPs were significantly enriched in skeletal muscle contraction, actin filament organization, myofibril assembly and positive regulation of myotube differentiation and so on in biological process (Figure 4A); and molecular function mainly included Ca^2+^ binding, tropomyosin binding, actin binding and microfilament motor activity (Figure 4B); in cellular component, these DEPs mainly belonged to Z disc, extracellular matrix, myosin complex and focal adhesion (Figure 4C). In addition, KEGG enrichment analysis showed that 312 DEPs were significantly enriched in regulation of actin cytoskeleton, focal adhesion, ECM‐receptor interaction and calcium signalling pathway (Figure 4D). Both GO and KEGG analysis suggested that the proteins which involved in skeletal muscle growth and development‐related signal pathways had been significantly changed after down‐regulation of lnc23, which further supported the important role of lnc23 in myogenic differentiation.

### 3.4 RNA pulldown captures lnc23 binding protein

**FIGURE 5:**
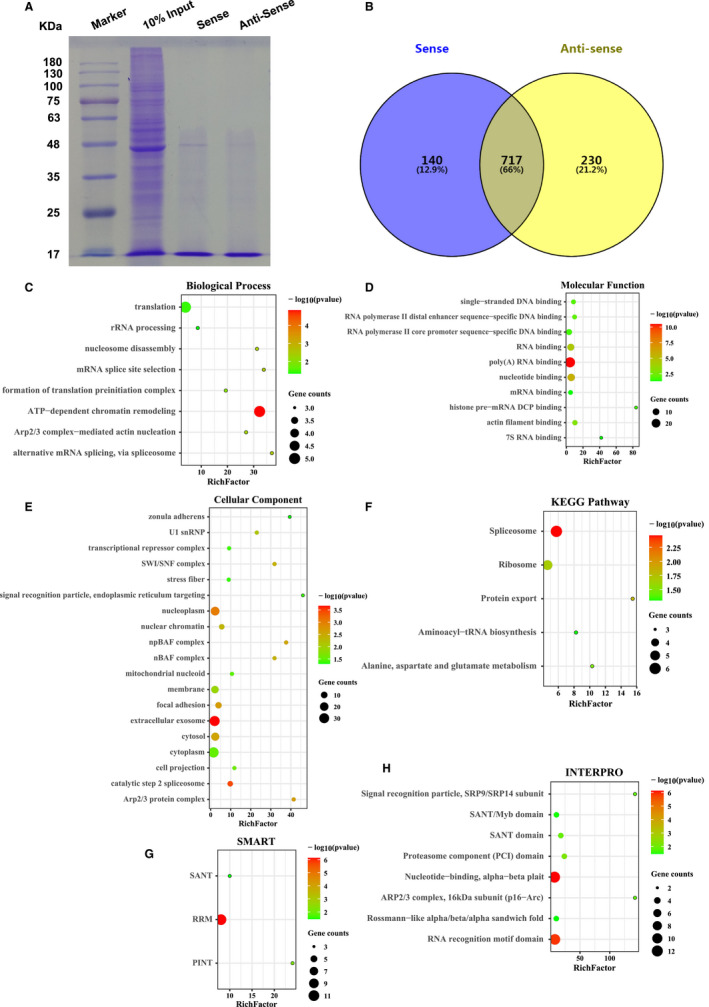
Identified proteins by RNA pulldown and their bioinformatics analysis. (A) SDS‐PAGE after RNA pulldown. 10% input and the anti‐sense chain of lnc23 were, respectively, as a positive control and a negative control. (B) Identified proteins by RNA pulldown/LC‐MS. A total of 857 proteins were identified from the sense chain of lnc23 and 947 proteins were identified from the anti‐sense chain of lnc23, of which 140 proteins specifically bound to the sense chain of lnc23. (C‐F) GO and KEGG analysis of lnc23 potential binding proteins by DAVID. The enrichment items or pathways were filtered with *P* < .05. For more items or pathways, only the top 20 with the most significant enrichment were displayed using R 3.63 software. (G‐H) Domain analysis of lnc23 potential binding proteins by DAVID. The enrichment items were filtered with *P* < .05. The significantly enriched items were displayed using R 3.63 software

Quantitative proteomics has found 312 potential regulatory proteins of lnc23. To delve into the mechanisms of lnc23 regulating myogenic differentiation, RNA pulldown was performed to narrow the study scope and obtain the proteins directly associated with lnc23. With 10% input and the anti‐sense chain of lnc23 were, respectively, as a positive control and a negative control, 12% SDS polyacrylamide gel electrophoresis (SDS‐PAGE) and coomassie blue staining showed that the proteins captured from the sense and anti‐sense chain of lnc23 had an evident difference at around 48 kD (Figure 5A). Then, the captured proteins were identified by LC‐MS. A total of 857 and 947 proteins were, respectively, identified from the sense and anti‐sense chain of lnc23, of which 140 proteins specifically bound to the sense chain of lnc23 and were deemed as lnc23 potential binding proteins (PBPs) (Figure 5B).

We also performed GO and KEGG analysis of lnc23 PBPs. Results exposed that lnc23 PBPs were remarkably enriched in ATP‐dependent chromatin remodelling, alternative mRNA splicing, Arp2/3 complex‐mediated actin nucleation and so on in biological process (Figure 5C). And molecular function mainly included poly (A) RNA binding, nucleotide binding, RNA binding and actin filament binding (Figure 5D). In cellular component, these proteins mainly belonged to extracellular exosome, focal adhesion and Arp2/3 protein complex (Figure 5E). Besides, KEGG enrichment analysis evinced lnc23 PBPs were markedly involved in spliceosome and protein export (Figure 5F). Synergistic analysed above results and quantitative proteomics, we suggested that lnc23 PBPs were involved in the process of myogenic differentiation through affecting cell fusion via Arp2/3 complex‐mediated actin nucleation. Furthermore, the domain analysis was employed using DAVID to determine the structural commonality of lnc23 PBPs and demonstrated that these proteins contained domains such as nucleotide‐binding domain and RNA recognition motif (RRM) domain (Figure 5G,H).

### 3.5 Analysis and identification of lnc23 candidate binding proteins

**FIGURE 6:**
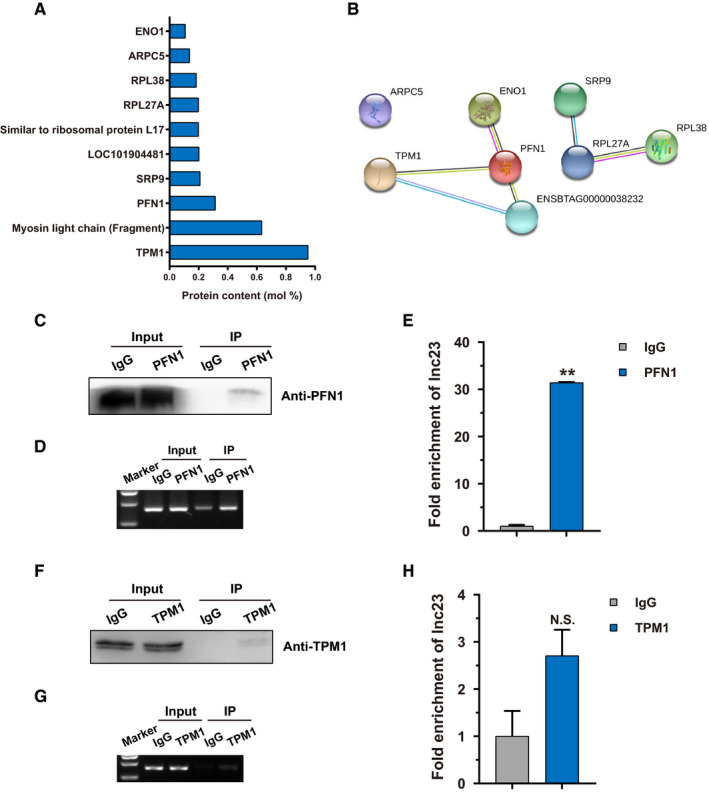
Screening and verifying of lnc23 binding proteins. (A) Candidate binding proteins of lnc23 at protein content (mol%) > 0.1%. (B) Protein‐protein interaction analysis of lnc23 candidate binding proteins by STRING. (C) Detection the IP efficiency of PFN1 by Western blot. 10% input and IgG were, respectively, as a positive control and a negative control. (D) Detection the enrichment of lnc23 in PFN1 immunoprecipitates by RT‐PCR. (E) Detection the fold enrichment of lnc23 in PFN1 immunoprecipitates by qRT‐PCR. (F) Detection the IP efficiency of TPM1 by Western blot. 10% input and IgG were, respectively, as a positive control and a negative control. (G) Detection the enrichment of lnc23 in TPM1 immunoprecipitates by RT‐PCR. (H) Detection the fold enrichment of lnc23 in TPM1 immunoprecipitates by qRT‐PCR

The interesting proteins were selected from lnc23 PBPs by the threshold of protein molar percentage more than 0.1%. A total of 10 proteins were screened as lnc23 candidate binding proteins (Figure 6A). PPI analysis of these 10 proteins showed that there was an interaction among TPM1, PFN1, LOC101904481 (ENSBTAG00000038232) and ENO1 (Figure 6B). Among them, TPM1 was a down‐regulated differential protein identified by quantitative proteomics, and its protein content was the highest in lnc23 PBPs. Therefore, comprehensively consider the results from quantitative proteomics, protein content and PPI analysis, TPM1 and PFN1 with high protein content and interaction with each other were selected as lnc23 candidate binding proteins for further study.

To determine the binding of lnc23 to TPM1 and PFN1, RIP experiment was performed for reverse verification. With higher immunoprecipitation efficiency, RT‐PCR and qRT‐PCR revealed that lnc23 was highly enriched in PFN1 immunoprecipitates, and the fold enrichment was 31.4 times compared with the negative control IgG (Figure 6C‐E). However, the immunoprecipitation efficiency of TPM1 was lower, and the fold enrichment of lnc23 also was low in TPM1 immunoprecipitates (Figure 6F‐H). In summary, we supported that PFN1, not TPM1, was a binding protein of lnc23. Therefore, the following study took PFN1 as the research object to continue exploring the synergistic regulation mechanisms of lnc23 and PFN1 in the myogenic differentiation of bovine skeletal muscle satellite cells.

### 3.6 Lnc23 accelerate myogenesis of bovine skeletal muscle satellite cells via PFN1

**FIGURE 7:**
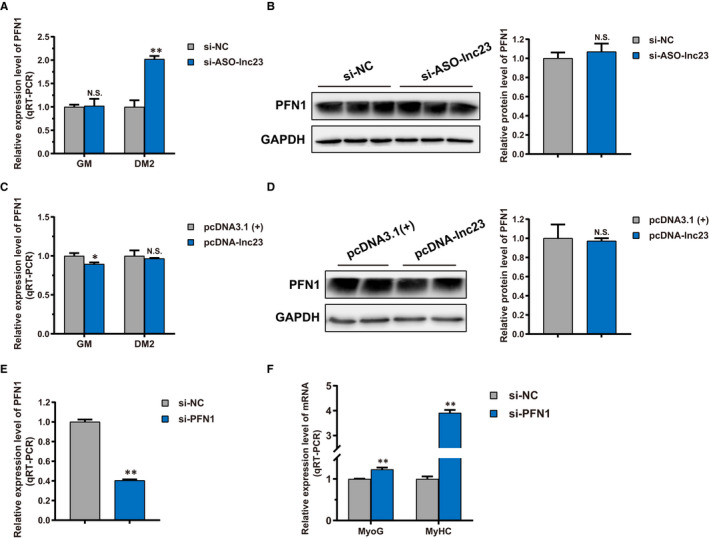
Effect of lnc23 on PFN1, and PFN1 on myogenic differentiation. (A) Down‐regulated lnc23 significantly elevated the mRNA expression level of PFN1 in DM2, (B) but did not affect the protein expression level of PFN1. (C) Up‐regulated lnc23 significantly decreased the mRNA expression level of PFN1 in GM (D); it also had no dramatic effect on the PFN1 protein. (E) Knockdown experiment resulted in a 60% decrease in PFN1 mRNA expression level (F) and produced a significant increase in mRNA expression level of cell differentiation‐related markers MyoG and MyHC in DM2

In previous quantitative proteomics, we found that down‐regulated lnc23 had no remarkable effect on the protein expression level of PFN1. To illuminate the mechanism of lnc23 cooperating with PFN1 in regulating myogenic differentiation, the effect of lnc23 on PFN1 was detected both at mRNA and protein levels, respectively. Down‐regulated lnc23 significantly elevated the mRNA expression level of PFN1 in DM2 (Figure 7A), but did not affect the protein expression level of PFN1 (Figure 7B). Reciprocally, up‐regulated lnc23 significantly decreased the mRNA expression level of PFN1 in GM (Figure 7C); it also had no dramatic effect on the PFN1 protein (Figure 7D). These results were consistent with the quantitative proteomics, which further proved the credibility of proteomics data. Hence, we speculated that lnc23 may affect the expression of PFN1 at transcriptional level, but not its translation. At the same time, we also investigated the effect of down‐regulating PFN1 on cell differentiation. With a 60% decrease in PFN1 mRNA (Figure 7E), the mRNA expression level of MyoG and MyHC was significantly elevated in DM2 (Figure 7F), indicating that PFN1 negatively regulated myogenic differentiation.

**FIGURE 8:**
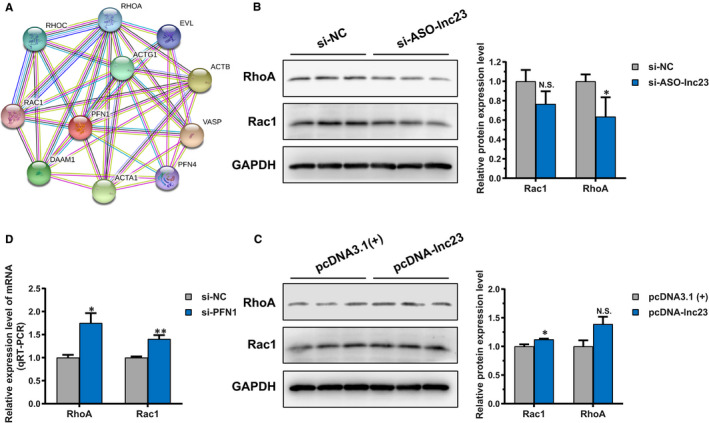
Effect of lnc23 on PFN1 interacting proteins. (A) Protein‐protein interaction (PPI) analysis of PFN1 interacting proteins by STRING. (B) Down‐regulated lnc23 inhibited the protein expression level of RhoA, while Rac1 protein level showed a descending trend. (C) Up‐regulated lnc23 promoted the protein expression level of Rac1, while RhoA protein level presented an elevated tendency. (D) The mRNA expression levels of RhoA and Rac1 were all significantly increased after down‐regulating PFN1. Note: The Western blot bands of RhoA and Rac1 came from the same polyvinylidene difluoride membrane as KBTBD10 and TPM1 in Figure S3, so GAPDH band was same

We have confirmed that lnc23 binds to PFN1, but does not affect its protein level. Given that, we posited that lnc23 might influence the binding of PFN1 interaction proteins. PPI analysis of PFN1 revealed that PFN1 interacted with RhoA, RhoC and Rac1 of small GTPase Rho family members (Figure 8A). Interestingly, RhoA and Rac1 played a prominent role in myogenic differentiation such as cell cycle, actin kinetics and cell fusion. Further study demonstrated that the protein level of RhoA was significantly decreased after down‐regulating lnc23, and the Rac1 protein showed a descending trend (Figure 8B). On the contrary, up‐regulated lnc23 produced a remarkable increase in Rac1 protein level and an elevated tendency on RhoA protein level (Figure 8C). Evidencing that lnc23 positively regulating the protein expression of RhoA and Rac1. Further study found that the mRNA levels of RhoA and Rac1 were drastically elevated after down‐regulating PFN1 (Figure 8D), indicating that PFN1 may negatively regulate the expression of its interacting proteins RhoA and Rac1. Above results illustrated that lnc23 could mediate the mRNA level of PFN1 to regulate the protein expression of RhoA and Rac1. Taken together, we speculated that lnc23 may reduce the inhibiting effect of PFN1 on RhoA and Rac1 by binding to PFN1, thereby promoting myogenic differentiation.

## 4 DISCUSSION

Accumulating evidence suggests that lncRNAs are emerging as a gatekeeper of the proliferation and differentiation of skeletal muscle satellite cells.^45^, ^46^, ^47^ However, lncRNAs have the characteristics of complex mechanism, diverse modes of action and low conservation. With positive regulation on bovine skeletal muscle satellite cells, lncMD is conservative in humans, mice and dogs, which belongs to a rare interspecific conservative lncRNA.^11^ A bovine muscle‐specific lncRNA lnc133b could promote cell proliferation and inhibit cell differentiation whether lnc133b was down‐regulated or up‐regulated.^48^ In current study, lnc23 was screened from high‐throughput sequencing of three representative periods of skeletal muscle development and found that down‐regulated lnc23 inhibited cell differentiation, while up‐regulated lnc23 promoted cell differentiation. Hence, we supported that lnc23 positively regulated the differentiation of bovine skeletal muscle satellite cells.

One of the mechanisms of lncRNAs is to exert regulatory effects by binding to proteins. For example, lncRNA MALAT1 competes with SIRT1 to bind DBC1, releasing SIRT1 and enhancing its deacetylation activity, deacetylating p53 and inhibiting the transcription of p53 downstream genes, thereby promoting cell proliferation and inhibiting cell apoptosis.^49^ Thus, we investigated the mechanism of lnc23 on regulating myogenic differentiation via analysing the proteins regulated by lnc23. Firstly, lnc23 was down‐regulated and the potential regulatory protein of lnc23 was analysed by quantitative proteomics. Then, lnc23 binding protein was captured by RNA pulldown technique and identified by mass spectrometry to screen the lnc23 candidate binding protein. The combination of lnc23 and its candidate binding protein was determined by RIP experiment. Finally, we explored the molecular mechanism of lnc23 and its binding proteins synergistically regulating the myogenic differentiation of bovine skeletal muscle satellite cells. Based on the results of quantitative proteomics and RNA pulldown, TPM1 and PFN1, which have high protein content and interaction with each other, were selected as interested proteins. In our study, TPM1 was identified as a down‐regulated protein by quantitative proteomics, and its protein content was the highest in lnc23 PBPs, which was considered the most likely binding protein of lnc23. Unexpectedly, RIP experiment showed that the fold enrichment of lnc23 in TPM1 immunoprecipitates was only 2.7 times compared with the negative control IgG, which may be due to the poor specificity of TPM1 polyclonal antibody. In addition, it also may be the difference between lysis conditions and physiological conditions resulting in false‐positive results in RNA pulldown experiment. Compared with TPM1, the fold enrichment of lnc23 in PFN1 immunoprecipitates was 31.4 times as much as IgG, suggesting that lnc23 binds to PFN1.

**FIGURE 9:**
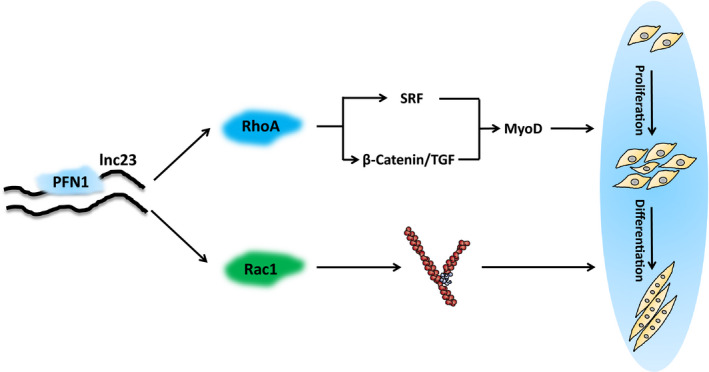
Potential mechanism model of lnc23 regulating proliferation and differentiation of bovine skeletal muscle satellite cells. Lnc23 may reduce the inhibited effect of PFN1 on RhoA and Rac1 by binding to PFN1, thereby promoting myogenic differentiation via the SRF and β‐catenin/TGF signalling pathway or the Arp2/3 complex‐mediated actin polymerization and cell fusion

Further studies demonstrated that lnc23 affected the mRNA expression level of PFN1 but not its protein level. And PFN1 negatively regulated the myogenic differentiation and its interaction proteins RhoA and Rac1 in mRNA level. In addition, lnc23 positively regulated the mRNA expression levels of RhoA and Rac1. It has been reported that RhoA positively regulates the expression of MyoD through SRF and β‐catenin/TGF, thereby promoting myogenic differentiation.^50^, ^51^ Interestingly, quantitative proteomics revealed that two subtypes of TGF, TGFB1 and TGFB2, were significantly down‐regulated after knocking down lnc23, which further suggesting lnc23 and its binding protein PFN1 may synergistically regulate the myogenic differentiation through RhoA mediated β‐catenin/TGF signalling pathway. Moreover, Arp2/3 regulates myogenic differentiation by mediating actin polymerization.^25^ And Rac1 mediates the polymerization of filamentous actin and results in lamellipodium formation and membrane ruffling at the leading edge of migrating cells.^33^ In addition, an experiment of Rac1 mutant mice has demonstrated that Rac1 promotes cell fusion by recruiting vinculin and cytoskeleton protein to the contact area between cells and cells, while the absence of Rac1 strongly suppresses the aggregation of Arp2/3 complex and myoblasts fusion.^41^ In our study, RNA pulldown captured ARPC5 was a subunit of Arp2/3 complex, which showed a descending tendency in quantitative proteomics although the difference was not significant. Meanwhile, ARPC1A was another subunit of Arp2/3 complex, which was significantly decreased in quantitative proteomics, suggesting that lnc23‐PFN1‐Rac1 may regulate myogenic differentiation through actin polymerization mediated by Arp2/3 complex. Taken together, we speculated that lnc23 may regulate the myogenesis of bovine skeletal muscle satellite cells mainly through two ways. Specifically, lnc23 weakens the inhibitory effect of PFN1 on RhoA by binding to PFN1 and promotes the expression of RhoA protein, thereby promoting myogenic differentiation by positively regulating the expression of MyoD through SRF and β‐catenin/TGF signalling pathways (Figure 9). Also, lnc23 attenuates the inhibitory effect of PFN1 on Rac1 by binding to PFN1 and promotes the expression of Rac1 protein, thereby promoting myogenic differentiation via accelerating actin polymerization and cell fusion mediated by Arp2/3 complex (Figure 9). However, it should be pointed out that lnc23‐PFN1‐RhoA/Rac1 axis is speculated based on the current research reports and experimental results, including quantitative proteomics, which is lack of certain experimental support. The detailed mechanism that lnc23 and PFN1 synergistically regulate these two signalling pathways would be verified in the future study.

More interestingly, the activity of small GTPase also is very critical for myogenic differentiation. The activity of Rac1 was high in proliferating myoblasts whereas decreased in differentiated myoblasts, further study indicating that Rac1 suppresses myogenic differentiation by preventing myoblasts from completely withdraw cell cycle.^52^, ^53^ Although the activity of RhoA is necessary for myogenic differentiation, the down‐regulation of RhoA activity is essential for the withdrawal of cell cycle, the expression of muscle differentiation genes and the myotube fusion.^54^, ^55^ Thus, to comprehensively delineate the synergistic mechanism between lnc23 and PFN1, we will also delve into the effect of lnc23 on small GTPase activity in the future.

Altogether, we discovered and identified a novel bovine muscle highly expressed lncRNA–lnc23, which could promote myogenic differentiation. Further study demonstrated that PFN1 was a binding protein of lnc23, and we initially elucidated that lnc23 might promote the myogenesis of bovine skeletal muscle satellite cells via PFN1‐RhoA/Rac1.

## 5 CONCLUSION

The novel identified lnc23 positively regulates the myogenesis of bovine skeletal muscle satellite cells via lnc23‐PFN1‐RhoA/Rac1 axis. In detail, lnc23 attenuates the inhibitory effect of PFN1 on RhoA/Rac1 by binding to PFN1 and promotes the protein expression of RhoA/Rac1, thereby accelerating myogenic differentiation. In short, lnc23 promotes skeletal muscle myogenesis via PFN1‐RhoA/Rac1. Nevertheless, there are still some limitations in this study. In general, this study is mainly focussed on the cellular level, our conclusion is just the tip of the iceberg, and the detailed mechanism of lnc23 cooperating with PFN1 in regulating myogenic differentiation is still a mystery. Thus, more evidence, especially animal experiments, is needed to support the current conclusion.

## CONFLICT OF INTEREST

The authors declare that they have no known competing financial interests or personal relationships that could have appeared to influence the work reported in this paper.

## AUTHOR CONTRIBUTIONS

**Mingming Chen:** Data curation (lead); methodology (equal); validation (lead); writing‐original draft (lead). **Linlin Zhang:** Data curation (equal); methodology (equal); validation (equal); writing‐review & editing (equal). **Yiwen Guo:** Methodology (equal); writing‐review & editing (equal). **Xinfeng Liu:** Methodology (equal); writing‐review & editing (equal). **Yingshen Song:** Data curation (supporting); validation (equal). **Xin Li:** Methodology (equal); writing‐review & editing (equal). **Xiangbin Ding:** Methodology (equal); writing‐review & editing (equal). **Hong Guo:** Methodology (equal); project administration (lead); writing‐review & editing (equal).

## Supporting information

Fig S1

Fig S2

Fig S3

Supplementary Material

## Data Availability

The data sets generated and analysed during the current study are not publicly available because of the data that also form part of an ongoing study, but are available from the corresponding author on reasonable request.

## References

[1] Berkes CA , Tapscott SJ . MyoD and the transcriptional control of myogenesis. Semin Cell Dev Biol. 2005;16:585‐595. 16099183 10.1016/j.semcdb.2005.07.006

[2] Buckingham M . Myogenic progenitor cells and skeletal myogenesis in vertebrates. Curr Opin Genet Dev. 2006;16:525‐532. 16930987 10.1016/j.gde.2006.08.008

[3] Braun T , Gautel M . Transcriptional mechanisms regulating skeletal muscle differentiation, growth and homeostasis. Nat Rev Mol Cell Biol. 2011;12:349‐361. 21602905 10.1038/nrm3118

[4] Mashinchian O , Pisconti A , Le Moal E , Bentzinger CF . The muscle stem cell niche in health and disease. Curr Top Dev Biol. 2018;126:23‐65. 29305000 10.1016/bs.ctdb.2017.08.003

[5] Sacco A , Doyonnas R , Kraft P , Vitorovic S , Blau HM . Self‐renewal and expansion of single transplanted muscle stem cells. Nature. 2008;456:502‐506. 18806774 10.1038/nature07384 PMC2919355

[6] Yin H , Price F , Rudnicki MA . Satellite cells and the muscle stem cell niche. Physiol Rev. 2013;93:23‐67. 23303905 10.1152/physrev.00043.2011 PMC4073943

[7] Zanou N , Gailly P . Skeletal muscle hypertrophy and regeneration: interplay between the myogenic regulatory factors (MRFs) and insulin‐like growth factors (IGFs) pathways. Cell Mol Life Sci. 2013;70:4117‐4130. 23552962 10.1007/s00018-013-1330-4 PMC11113627

[8] Hernandez‐Torres F , Rodriguez‐Outeirino L , Franco D , Aranega AE . Pitx2 in embryonic and adult myogenesis. Front Cell Dev Biol. 2017;5:46. 28507987 10.3389/fcell.2017.00046 PMC5410577

[9] Li Y , Chen X , Sun H , Wang H . Long non‐coding RNAs in the regulation of skeletal myogenesis and muscle diseases. Cancer Lett. 2018;417:58‐64. 29253523 10.1016/j.canlet.2017.12.015

[10] Simionescu‐Bankston A , Kumar A . Noncoding RNAs in the regulation of skeletal muscle biology in health and disease. J Mol Med (Berl). 2016;94:853‐866. 27377406 10.1007/s00109-016-1443-y PMC4957971

[11] Sun X , Li M , Sun Y , et al. The developmental transcriptome sequencing of bovine skeletal muscle reveals a long noncoding RNA, lncMD, promotes muscle differentiation by sponging miR‐125b. Biochim Biophys Acta. 2016;1863:2835‐2845. 27589905 10.1016/j.bbamcr.2016.08.014

[12] Wang L , Zhao Y , Bao X , et al. LncRNA Dum interacts with Dnmts to regulate Dppa2 expression during myogenic differentiation and muscle regeneration. Cell Res. 2015;25:335‐350. 25686699 10.1038/cr.2015.21 PMC4349245

[13] Blin‐Wakkach C , Lezot F , Ghoul‐Mazgar S , et al. Endogenous Msx1 antisense transcript: in vivo and in vitro evidences, structure, and potential involvement in skeleton development in mammals. Proc Natl Acad Sci U S A. 2001;98:7336‐7341. 11390985 10.1073/pnas.131497098 PMC34669

[14] Cai B , Li Z , Ma M , et al. LncRNA‐Six1 encodes a micropeptide to activate Six1 in Cis and is involved in cell proliferation and muscle growth. Front Physiol. 2017;8:230. 28473774 10.3389/fphys.2017.00230 PMC5397475

[15] Cabili MN , Trapnell C , Goff L , et al. Integrative annotation of human large intergenic noncoding RNAs reveals global properties and specific subclasses. Genes Dev. 2011;25:1915‐1927. 21890647 10.1101/gad.17446611 PMC3185964

[16] Billerey C , Boussaha M , Esquerre D , et al. Identification of large intergenic non‐coding RNAs in bovine muscle using next‐generation transcriptomic sequencing. BMC Genom. 2014;15:499. 10.1186/1471-2164-15-499 PMC4073507 24948191

[17] Koufariotis LT , Chen YP , Chamberlain A , Vander Jagt C , Hayes BJ . A catalogue of novel bovine long noncoding RNA across 18 tissues. PLoS One. 2015;10:e0141225. 26496443 10.1371/journal.pone.0141225 PMC4619662

[18] Liu XF , Ding XB , Li X , et al. An atlas and analysis of bovine skeletal muscle long noncoding RNAs. Anim Genet. 2017;48:278‐286. 28262958 10.1111/age.12539

[19] Yue B , Li H , Liu M , et al. Characterization of lncRNA‐miRNA‐mRNA network to reveal potential functional ceRNAs in bovine skeletal muscle. Front Genet. 2019;10:91. 30842787 10.3389/fgene.2019.00091 PMC6391848

[20] Behnen M , Murk K , Kursula P , et al. Testis‐expressed profilins 3 and 4 show distinct functional characteristics and localize in the acroplaxome‐manchette complex in spermatids. BMC Cell Biol. 2009;10:34. 19419568 10.1186/1471-2121-10-34 PMC2694148

[21] Machesky LM , Cole NB , Moss B , Pollard TD . Vaccinia virus expresses a novel profilin with a higher affinity for polyphosphoinositides than actin. Biochemistry. 1994;33:10815‐10824. 8075084 10.1021/bi00201a032

[22] Hu E , Chen Z , Fredrickson T , Zhu Y . Molecular cloning and characterization of profilin‐3: a novel cytoskeleton‐associated gene expressed in rat kidney and testes. Exp Nephrol. 2001;9:265‐274. 11423726 10.1159/000052621

[23] Boukhelifa M , Moza M , Johansson T , et al. The proline‐rich protein palladin is a binding partner for profilin. FEBS J. 2006;273:26‐33. 16367745 10.1111/j.1742-4658.2005.05036.x

[24] Romeo GR , Moulton KS , Kazlauskas A . Attenuated expression of profilin‐1 confers protection from atherosclerosis in the LDL receptor null mouse. Circ Res. 2007;101:357‐367. 17615372 10.1161/CIRCRESAHA.107.151399

[25] Richardson BE , Beckett K , Nowak SJ , Baylies MK . SCAR/WAVE and Arp2/3 are crucial for cytoskeletal remodeling at the site of myoblast fusion. Development. 2007;134:4357‐4367. 18003739 10.1242/dev.010678 PMC2880884

[26] Machesky LM , Goldschmidt‐Clermont PJ , Pollard TD . The affinities of human platelet and Acanthamoeba profilin isoforms for polyphosphoinositides account for their relative abilities to inhibit phospholipase C. Cell Regul. 1990;1:937‐950. 1966040 10.1091/mbc.1.12.937 PMC362863

[27] Evans NJ , Walker JW . Endothelin‐1 mobilizes profilin‐1‐bound PIP2 in cardiac muscle. Exp Biol Med (Maywood). 2006;231:882‐887. 16741017

[28] Stroud MJ , Feng W , Zhang J , et al. Nesprin 1alpha2 is essential for mouse postnatal viability and nuclear positioning in skeletal muscle. J Cell Biol. 2017;216:1915‐1924. 28533284 10.1083/jcb.201612128 PMC5496623

[29] Henty‐Ridilla JL , Juanes MA , Goode BL . Profilin directly promotes microtubule growth through residues mutated in amyotrophic lateral sclerosis. Curr Biol. 2017;27(22):3535‐3543.e4. 29129529 10.1016/j.cub.2017.10.002 PMC5772683

[30] Duan R , Gallagher PJ . Dependence of myoblast fusion on a cortical actin wall and nonmuscle myosin IIA. Dev Biol. 2009;325:374‐385. 19027000 10.1016/j.ydbio.2008.10.035 PMC2823627

[31] Fulton AB , Prives J , Farmer SR , Penman S . Developmental reorganization of the skeletal framework and its surface lamina in fusing muscle cells. J Cell Biol. 1981;91:103‐112. 7197679 10.1083/jcb.91.1.103 PMC2111955

[32] Jaffe AB , Hall A . Rho GTPases: biochemistry and biology. Annu Rev Cell Dev Biol. 2005;21:247‐269. 16212495 10.1146/annurev.cellbio.21.020604.150721

[33] Bryan BA , Li D , Wu X , Liu M . The Rho family of small GTPases: crucial regulators of skeletal myogenesis. Cell Mol Life Sci. 2005;62:1547‐1555. 15905962 10.1007/s00018-005-5029-z PMC11139171

[34] Sinha S , Yang W . Cellular signaling for activation of Rho GTPase Cdc42. Cell Signal. 2008;20:1927‐1934. 18558478 10.1016/j.cellsig.2008.05.002

[35] Etienne‐Manneville S , Hall A . Rho GTPases in cell biology. Nature. 2002;420:629‐635. 12478284 10.1038/nature01148

[36] Spiering D , Hodgson L . Dynamics of the Rho‐family small GTPases in actin regulation and motility. Cell Adh Migr. 2011;5:170‐180. 21178402 10.4161/cam.5.2.14403 PMC3084983

[37] Pirone DM , Liu WF , Ruiz SA , et al. An inhibitory role for FAK in regulating proliferation: a link between limited adhesion and RhoA‐ROCK signaling. J Cell Biol. 2006;174:277‐288. 16847103 10.1083/jcb.200510062 PMC2064187

[38] Mammoto A , Huang S , Moore K , Oh P , Ingber DE . Role of RhoA, mDia, and ROCK in cell shape‐dependent control of the Skp2‐p27kip1 pathway and the G1/S transition. J Biol Chem. 2004;279:26323‐26330. 15096506 10.1074/jbc.M402725200

[39] Hill CS , Wynne J , Treisman R . The Rho family GTPases RhoA, Rac1, and CDC42Hs regulate transcriptional activation by SRF. Cell. 1995;81:1159‐1170. 7600583 10.1016/s0092-8674(05)80020-0

[40] Gauthier‐Rouviere C , Vandromme M , Tuil D , et al. Expression and activity of serum response factor is required for expression of the muscle‐determining factor MyoD in both dividing and differentiating mouse C2C12 myoblasts. Mol Biol Cell. 1996;7:719‐729. 8744946 10.1091/mbc.7.5.719 PMC275925

[41] Vasyutina E , Martarelli B , Brakebusch C , Wende H , Birchmeier C . The small G‐proteins Rac1 and Cdc42 are essential for myoblast fusion in the mouse. Proc Natl Acad Sci U S A. 2009;106:8935‐8940. 19443691 10.1073/pnas.0902501106 PMC2682539

[42] Dai Y , Wang YM , Zhang WR , et al. The role of microRNA‐1 and microRNA‐206 in the proliferation and differentiation of bovine skeletal muscle satellite cells. Vitro Cell Dev Biol Anim. 2016;52:27‐34. 10.1007/s11626-015-9953-4 26424132

[43] Chen M , Li X , Zhang X , et al. A novel long non‐coding RNA, lncKBTBD10, involved in bovine skeletal muscle myogenesis. Vitro Cell Dev Biol Anim. 2019;55:25‐35. 10.1007/s11626-018-0306-y 30465303

[44] Shinoda K , Tomita M , Ishihama Y . emPAI Calc–for the estimation of protein abundance from large‐scale identification data by liquid chromatography‐tandem mass spectrometry. Bioinformatics. 2010;26:576‐577. 20031975 10.1093/bioinformatics/btp700

[45] Hitachi K , Nakatani M , Takasaki A , et al. Myogenin promoter‐associated lncRNA Myoparr is essential for myogenic differentiation. EMBO Rep. 2019;20. 10.15252/embr.201847468 PMC6399612 30622218

[46] Militello G , Hosen MR , Ponomareva Y , et al. A novel long non‐coding RNA Myolinc regulates myogenesis through TDP‐43 and Filip1. J Mol Cell Biol. 2018;10:102‐117. 29618024 10.1093/jmcb/mjy025 PMC7191624

[47] Tajbakhsh S . lncRNA‐encoded polypeptide SPAR(s) with mTORC1 to regulate skeletal muscle regeneration. Cell Stem Cell. 2017;20:428‐430. 28388426 10.1016/j.stem.2017.03.016

[48] Jin CF , Li Y , Ding XB , et al. lnc133b, a novel, long non‐coding RNA, regulates bovine skeletal muscle satellite cell proliferation and differentiation by mediating miR‐133b. Gene. 2017;630:35‐43. 28757453 10.1016/j.gene.2017.07.066

[49] Chen R , Liu Y , Zhuang H , et al. Quantitative proteomics reveals that long non‐coding RNA MALAT1 interacts with DBC1 to regulate p53 acetylation. Nucleic Acids Res. 2017;45:9947‐9959. 28973437 10.1093/nar/gkx600 PMC5622371

[50] Gopinath SD , Narumiya S , Dhawan J . The RhoA effector mDiaphanous regulates MyoD expression and cell cycle progression via SRF‐dependent and SRF‐independent pathways. J Cell Sci. 2007;120:3086‐3098. 17684061 10.1242/jcs.006619

[51] L'Honore A , Rana V , Arsic N , Franckhauser C , Lamb NJ , Fernandez A . Identification of a new hybrid serum response factor and myocyte enhancer factor 2‐binding element in MyoD enhancer required for MyoD expression during myogenesis. Mol Biol Cell. 2007;18:1992‐2001. 17377068 10.1091/mbc.E06-09-0867 PMC1877109

[52] Heller H , Gredinger E , Bengal E . Rac1 inhibits myogenic differentiation by preventing the complete withdrawal of myoblasts from the cell cycle. J Biol Chem. 2001;276:37307‐37316. 11489882 10.1074/jbc.M103195200

[53] Travaglione S , Messina G , Fabbri A , et al. Cytotoxic necrotizing factor 1 hinders skeletal muscle differentiation in vitro by perturbing the activation/deactivation balance of Rho GTPases. Cell Death Differ. 2005;12:78‐86. 15514676 10.1038/sj.cdd.4401522

[54] Charrasse S , Comunale F , Grumbach Y , Poulat F , Blangy A , Gauthier‐Rouviere C . RhoA GTPase regulates M‐cadherin activity and myoblast fusion. Mol Biol Cell. 2006;17:749‐759. 16291866 10.1091/mbc.E05-04-0284 PMC1356585

[55] Iwasaki K , Hayashi K , Fujioka T , Sobue K . Rho/Rho‐associated kinase signal regulates myogenic differentiation via myocardin‐related transcription factor‐A/Smad‐dependent transcription of the Id3 gene. J Biol Chem. 2008;283:21230‐21241. 18477564 10.1074/jbc.M710525200 PMC3258938

