# Supplementary figures and images for: A novel lncRNA promotes myogenesis of bovine skeletal muscle satellite cells via PFN1‐RhoA/Rac1
Source: J Cell Mol Med. 2021 May 4;25(13):5988–6005. doi: 10.1111/jcmm.16427 (PMC8256363; doi:10.1111/jcmm.16427)

**A**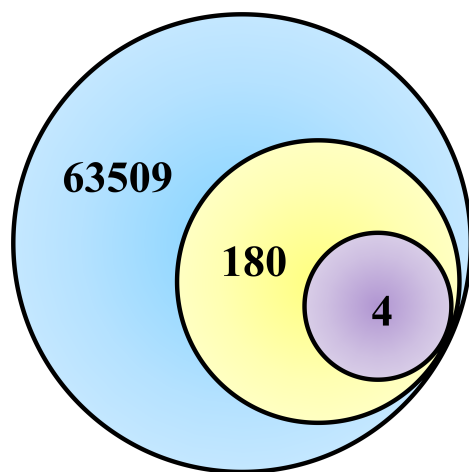

- 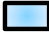 Total lncRNAs
- 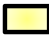 Co-expressed lncRNAs
- 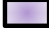 RPKM > 5

**B**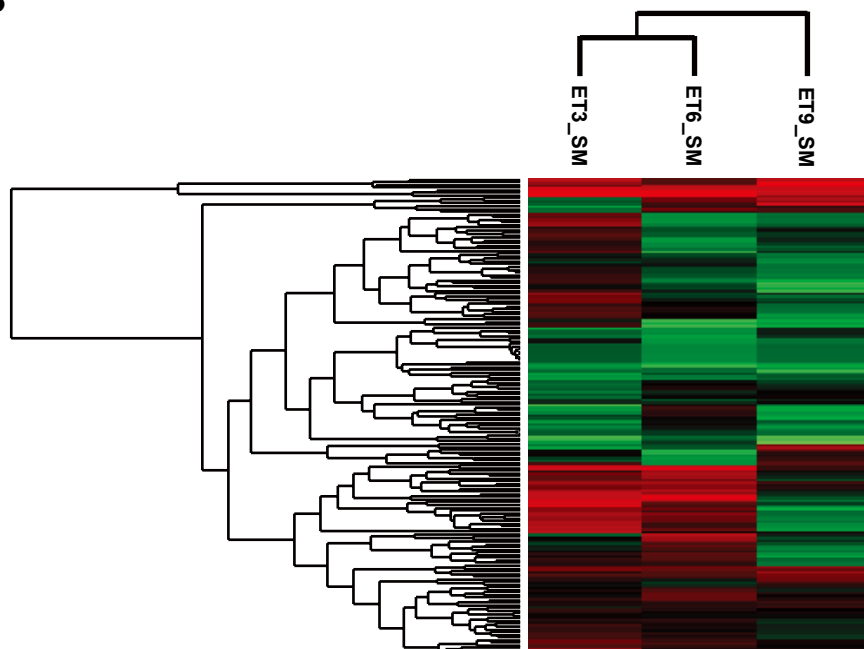

Supplement: Supplementary file 1 — Fig S1 [file JCMM-25-5988-s002.pdf]

**A**

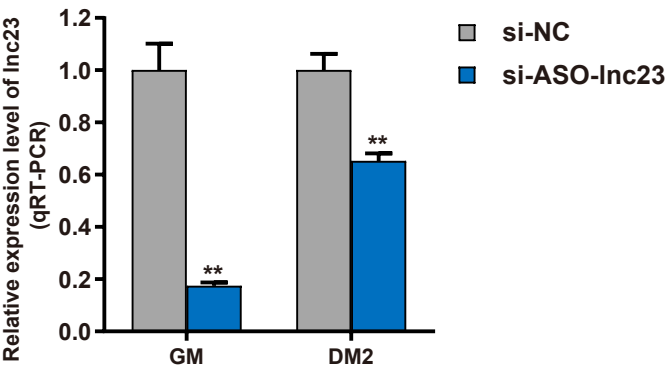

**B**

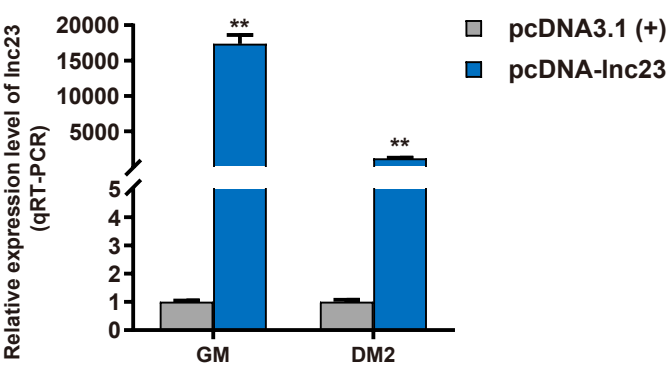

**C**

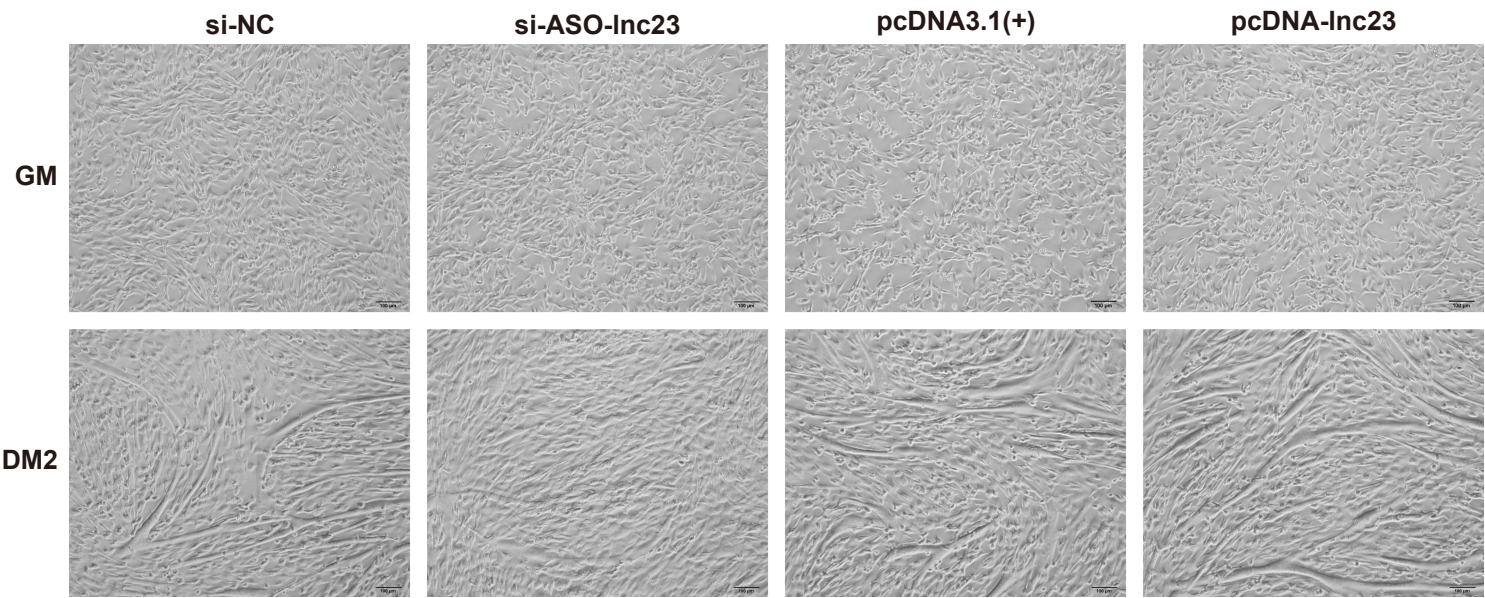

Supplement: Supplementary file 2 — Fig S2 [file JCMM-25-5988-s004.pdf]

**A**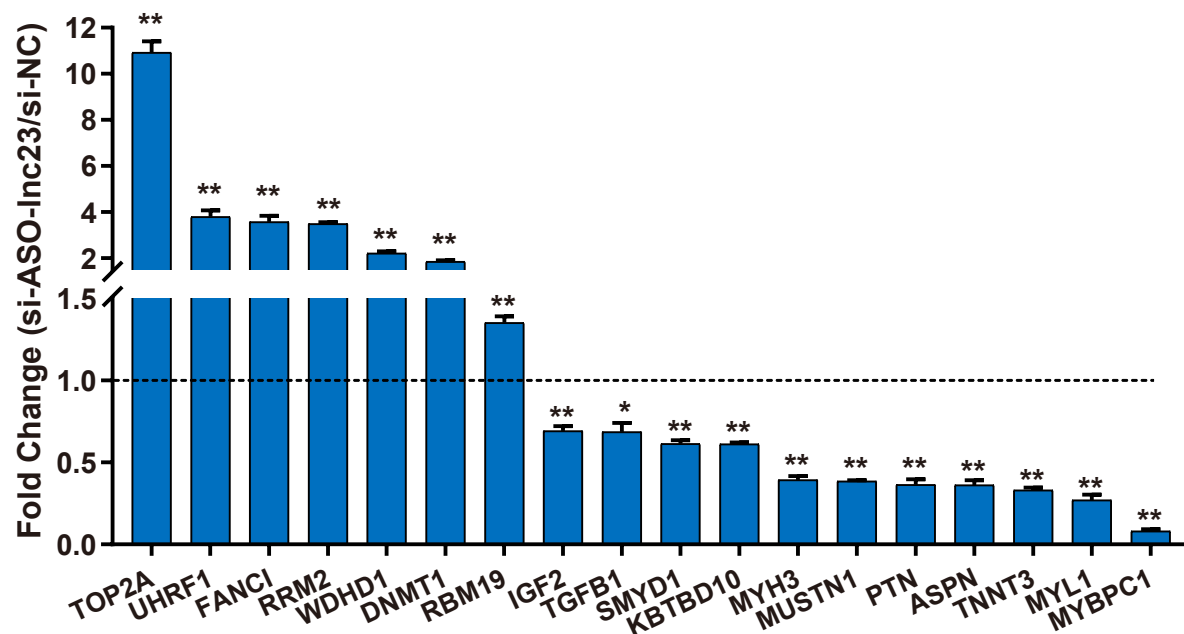**B**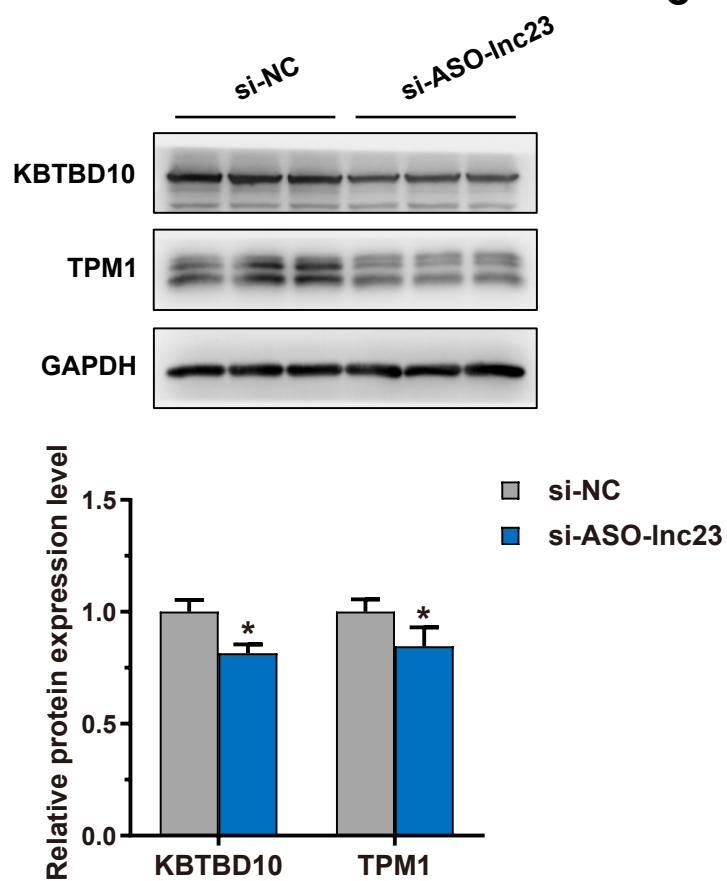**C**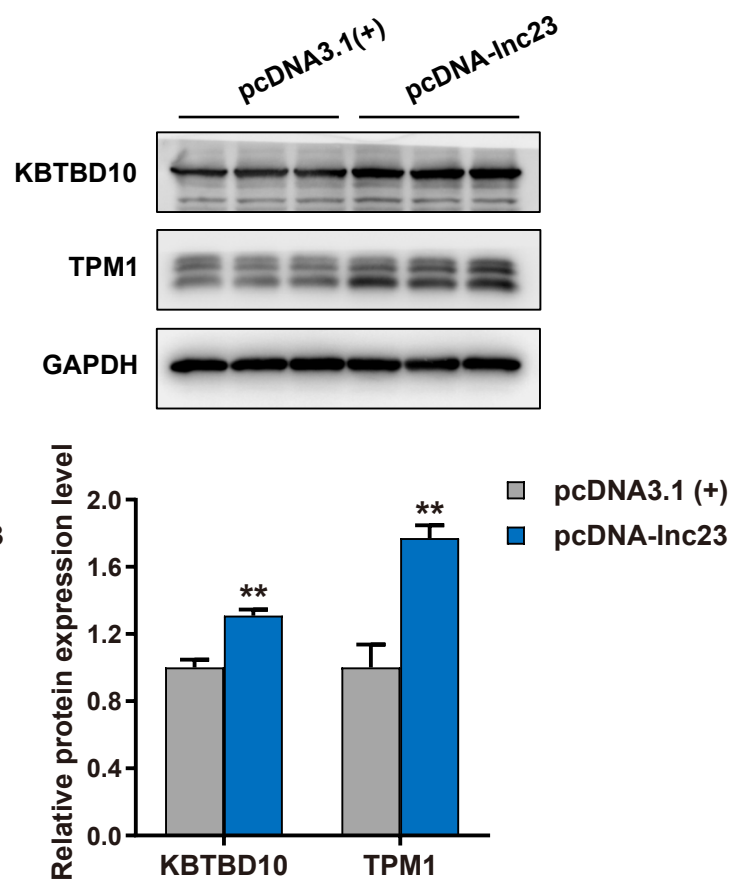

Supplement: Supplementary file 3 — Fig S3 [file JCMM-25-5988-s003.pdf]
