# Supplementary material for: A novel lncRNA promotes myogenesis of bovine skeletal muscle satellite cells via PFN1‐RhoA/Rac1
Source: J Cell Mol Med. 2021 May 4;25(13):5988–6005. doi: 10.1111/jcmm.16427 (PMC8256363; doi:10.1111/jcmm.16427)
Supplement: Supplementary file 4 — Supplementary Material [file JCMM-25-5988-s001.docx]

# A novel lncRNA promotes myogenesis of bovine skeletal muscle satellite cells via PFN1-RhoA/Rac1

Mingming Chen^#^, Linlin Zhang^#^, Yiwen Guo, Xinfeng Liu, Yingshen Song, Xin Li, Xiangbin Ding, Hong Guo^*^

Tianjin Key Laboratory of Agricultural Animal Breeding and Healthy Husbandry, College of Animal Science and Veterinary Medicine, Tianjin Agricultural University, Tianjin 300384, China

^#^ These authors contributed equally to the work

* Corresponding authors

Address: NO.22 Jinjing Road, Xiqing District, Tianjin City, Tianjin 300384, China

Tel: +86 22 2378-1297

Fax: +86 22 2378-4590

E-mail address: [guohong64@163.com](mailto:guohong64@163.com)

**Supplementary Figure 1 Analysis of sequencing results and screening of candidate lncRNAs**

(A) Statistics of total lncRNAs, co-expressed lncRNAs and the screening of high abundance lncRNAs. (B) Cluster analysis of co-expression of lncRNAs in muscle tissue of three representative periods.

**Supplementary Figure 2 Lnc23 loss-of-function and gain-of-function model and its effect on cell differentiation**

(A) Knockdown of lnc23 significantly reduced its expression level in GM and DM2. (B) Over-expression of lnc23 produced more than 17,367-fold and 1,144-fold increase of lnc23 in GM and DM2.

(C) Knockdown of lnc23 inhibited the differentiation process of bovine skeletal muscle satellite cells, while over-expression of lnc23 promoted differentiation process of bovine skeletal muscle satellite cells (scale bars 100 μm).

**Supplementary Figure 3 Verification of differential expressed proteins of TMT 10-plex labeling quantitative proteomics**

(A) Verification of differential expressed proteins at mRNA level when lnc23 was down regulated in DM2 by qRT-PCR. The ratio of si-ASO-lnc23/si-NC was as fold change, and more than 1.0 was considered as up-regulation, while less than 1.0 was considered as down-regulation. (B) Verification of differential expressed proteins at protein level after lnc23 was knocked down and over-expressed in DM2 by Western blot. NOTE: The western blot bands of KBTBD10 and TPM1 came from the same polyvinylidene difluoride membrane as RhoA and Rac1 in Figure 8, so GAPDH band was same.

**Supplementary Table 1 Primers of qRT-PCR**

| Genes |  | Primer sequences（5’-3’） | Product length (bp) |
| --- | --- | --- | --- |
| GAPDH | F | TGTTGTGGATCTGACCTGCC | 135 |
|  | R | AAGTCGCAGGAGACAACCTG |  |
| pre-GAPDH | F | TCCCTCCACGATGCCAAAG | 143 |
|  | R | GAAGCGGGCTGATAGTGCC |  |
| lnc23 | F | CTCCTTCTGCCCTGTAGTCA | 165 |
|  | R | AGTACTGCCCTGATTGCTGA |  |
| MyoG | F | GGCTGACAAATGCCAGACTATCC | 140 |
|  | R | TGGTCCCTTGCTTTATCTCCCT |  |
| MyHC | F | CTGGAATCCGGAGGCAGAA | 105 |
|  | R | TTTTCGAAGGTAGGGAGCGG |  |
| TOP2A | F | TGAATAACAGCAGAAACCGTCA | 89 |
|  | R | GCAATCTGAGGGGACAAGTCA |  |
| RRM2 | F | CAGCTTAGCGGACAAGGAGA | 144 |
|  | R | AGAAGTGGTTCGTCCTCCAC |  |
| DNMT1 | F | GACTCCACCTACGAAGACCTGAT | 124 |
|  | R | CTACTTGCTCCACCACGAACTG |  |
| WDHD1 | F | TGTGGAACTTGCTGGTCTAATGA | 129 |
|  | R | GCCAATTCTGCTGCCTTCTCTA |  |
| RBM19 | F | CGTGTTACCATCCACCATCAAGA | 130 |
|  | R | CAGCGTGTTCCAGTTGTGAGA |  |
| PTN | F | ATCTGAACACGGCTCTGAAGAC | 126 |
|  | R | CTGCTTGAGGTTTGGACTTGGT |  |
| MYBPC1 | F | TATGTGCGTGTGAAGGCTGTG | 146 |
|  | R | TCTCCAACTCTGCGGATATAGGT |  |
| TNNT3 | F | AGCTGGAGACCGACAAGTTC | 95 |
|  | R | CTTCTGGGCCTGGTCGATG |  |
| MUSTN1 | F | AAGACCTACCAGGTCATGCG | 102 |
|  | R | GGGCTTCTCAAACACGGTCT |  |
| MYL1 | F | AGAAGTGGAAGCGCTGATGG | 119 |
|  | R | AGGGAACAGATAGATACCTTGAGA |  |
| ASPN | F | GATTTCTGCCCAACAGTGCC | 96 |
|  | R | TGCAGGTTGTACCTCCCAAT |  |
| MYH3 | F | TGAACGCCCTCTCCAAATCC | 101 |
|  | R | AATGAAGTGCTGTCTCGGCA |  |
| SMYD1 | F | AACATCTACACGCTGCGACTG | 104 |
|  | R | TAGCCATCCACCATCCTCCTG |  |
| TGFB1 | F | ACCTGGCACGGTGTTTGTAA | 128 |
|  | R | CAGGGTCACACAGAAGGGTC |  |
| IGF2 | F | CGTGGCATCGTGGAAGAGTG | 109 |
|  | R | CGGTCGTAGAGGCAGACACAT |  |
| UHRF1 | F | CTGTAAGGACGATGAGCGGAAG | 125 |
|  | R | GCAGGCAGTAGATGTGGAAGG |  |
| FANCI | F | AAACAGCTGCCCCTACTGTC | 150 |
|  | R | GGTTTGTTAGGGCTGCCTGA |  |
| KBTBD10 | F | TGGTGAGGTGAACGGTGATG | 140 |
|  | R | TTTCCGTGGGATCGTAAGCC |  |
| PFN1 | F | GTGGAGCCCCAACCTTCAAT | 94 |
|  | R | TTGATCATACCGCCGTGGAC |  |
| RhoA | F | GATGTCCAACCCACCTGACC | 92 |
|  | R | AATTAGCGCCTGGTGTGTCA |  |
| Rac1 | F | TCCCAACACACCCATCATCCT | 93 |
|  | R | GGCGTCAGCTTCTTCTCCTTC |  |

**Supplementary Table 2 Primers of 5’-RACE PCR**

| Primer Name | Primer sequences（5’-3’） | Tm (℃) | GC (%) |
| --- | --- | --- | --- |
| GSP1-A | CCACTGACTACAGGGCAGAAGGAGGGG | 69.4 | 63.0 |
| GSP1-B | TCCACTGACTACAGGGCAGAAGGAGGGG | 70.2 | 61.0 |

**Supplementary Table 3 Walking primers of 3'-RACE PCR**

| Genes |  | Primer sequences（5’-3’） | Tm (℃) | GC (%) |
| --- | --- | --- | --- | --- |
| GSP2-A | F | TTCTGCCCTGTAGTCAGTGGAT | 60.8 | 50.0 |
|  | R | GCCTCGGTGAACTCCAGAATA | 59.5 | 52.4 |
| GSP2-B | F | GCATCTACCCTGGGAGTCTGA | 60.7 | 57.1 |
|  | R | AGATCACCCATTTGTCGTCATG | 58.7 | 45.5 |
| GSP2-C | F | CTAGGGGATTTCTAGGCTCTGC | 60.7 | 54.6 |
|  | R | CCCCTCCACAAACAGCAATCTC | 65.2 | 54.6 |

**Supplementary Table 4 Primers sequences of gene cloning**

| Genes | Primer Name | Primer Sequences（5’-3’） |
| --- | --- | --- |
| FSH-β | FSH-β-F | CGGAATTCATGAAGTCTGTCCAGTTCTGTTTCC |
|  | FSH-β-R | CCCAAGCTTTTATTCTTTGATTTCCCTGAAGGAG |
| lnc23 (+) | Bta-EcoRV-F | CCGATATCGGGAGGAGAGACAGAGCGAC |
|  | Bta-XhoI-R | CCGCTCGAGCCCTCCACAAACAGCAATCTC |
| lnc23 (-) | Bta-anti-EcoRV-F | CCGATATCCCCTCCACAAACAGCAATCTC |
|  | Bta-anti-XhoI-R | CCGCTCGAGGGGAGGAGAGACAGAGCGAC |
| lnc23 | lnc23-BM-F | gcggcagccatatggctagcGGGAGGAGAGACAGAGCGACCA |
|  | lnc23-BM-R | tgtccaccagtcatgctagcCCCTCCACAAACAGCAATCTCT |

Note: lnc23-BM-F and lnc23-BM-R are homologous recombination primers of lnc23 and pET-28a. The underlined position is the restriction enzyme cutting site in the table.

**Supplementary Table 5 Sequences of si-ASO-lnc23 and si-PFN1**

| Fragment Name | Sequence (5’-3’) |
| --- | --- |
| ASO-bta-lnc23_001 | GAATGGCCTCAGAAAGACTC |
| ASO-bta-lnc23_002 | GAGATAACACATGTGGAGGG |
| ASO-bta-lnc23_003 | CATAGAACAGGCTGATGGCC |
| si-bta-lnc23_001 | CCACCACAGAGTGGGTTAA |
| si-bta-lnc23_002 | GGAGGGATGTTGTGTCACA |
| si-bta-lnc23_003 | GCATTCAGAACTTAAGTAA |
| si-bta-PFN1_001 | GCGGTATGATCAACAAGAA |
| si-bta-PFN1_002 | GCAAAGACCGGTCAAGTTT |
| si-bta-PFN1_003 | CCGGCAAGACCTTCGTCAA |

**Supplementary Table 6 The antibodies information**

| Antibody Name | Manufacturer | Catalog Number | Host | Final Concentration |
| --- | --- | --- | --- | --- |
| MyoG | DSHB | F5D | Mouse | 1:100 |
| GAPDH | Zhongshan Golden Bridge | TA-08 | Mouse | 1:1000 |
| KBTBD10 | abcam | ab66605 | Rabbit | 1:1000 |
| TPM1 (TM311) | Novus Biologicals | NB100-1908 | Mouse | 1:1000 |
| RhoA | NewEast | 26007 | Mouse | 1:1000 |
| Rac1 | NewEast | 26005 | Mouse | 1:1000 |
| PFN1 | abcam | ab50667 | Rabbit | 1.0 μg/mL |
| MyHC | DSHB | MF20 | Mouse | 0.5 μg/mL |
